# Semi-Crystalline Hydrophobic Polyamidoamines: A New Family of Technological Materials?

**DOI:** 10.3390/polym13071018

**Published:** 2021-03-25

**Authors:** Massimo Marcioni, Jenny Alongi, Elisabetta Ranucci, Mario Malinconico, Paola Laurienzo, Paolo Ferruti, Amedea Manfredi

**Affiliations:** 1 Dipartimento di Chimica, Università Degli Studi di Milano, via C. Golgi 19, 20133 Milano, Italy; massimo.marcioni@polito.it (M.M.); jenny.alongi@unimi.it (J.A.); elisabetta.ranucci@unimi.it (E.R.); 2 Dipartimento di Scienza Applicata e Tecnologia, Politecnico di Torino, Alessandria Campus, Viale T. Michel, 15121 Alessandria, Italy; 3 Istituto Polimeri, Compositi e Biomateriali (IPCB), Consiglio Nazionale Delle Ricerche, via Campi Flegrei 34, 80078 Pozzuoli, Italy; mario.malinconico@ipcb.cnr.it (M.M.); paola.laurienzo@ipcb.cnr.it (P.L.)

**Keywords:** linear polyamidoamines, semi-crystalline polyamidoamines, hydrophobic polyamidoamines, flame resistant polymers

## Abstract

The hitherto known polyamidoamines (PAAs) are not suitable as structural materials because they are usually water-soluble or swellable in water. This paper deals with the synthesis and characterization of semi-crystalline hydrophobic PAAs (H-PAAs) by combining different bis-*sec*-amines with bis-acrylamides obtained from C6–C12 bis-*prim*-amines. H-PAAs were initially obtained in a solution of benzyl alcohol, a solvent suitable for both monomers and polymers. Their number average molecular weights, ${\bar{M}}_{n}$, which were determined with ^1^H-NMR by evaluating the percentage of their terminal units, varied from 6000 to >10,000. The solubility, thermal properties, ignitability and water resistance of H-PAAs were determined. They were soluble in organic solvents, semi-crystalline and thermally stable. The most promising ones were also prepared using a bulk process, which has never been previously reported for PAA synthesis. In the form of films, these H-PAAs were apparently unaffected by water. The films underwent tensile and wettability tests. They showed similar Young moduli (260–263 MPa), whereas the maximum stress and the stress at break depended on the number of methylene groups of the starting bis-acrylamides. Their wettability was somewhat higher than that of common Nylons. Interestingly, none of the H-PAAs considered, either as films or powders, ignited after prolonged exposure to a methane flame.

## 1. Introduction

Linear polyamidoamines (PAAs) are synthetic polymers obtained from the aza-Michael polyaddition of *prim*-monoamines or bis-*sec*-amines with bis-acrylamides [1,2,3]. The reaction is specific since other chemical functions, except thiols and *prim*- or *sec*-phosphine groups, do not participate in it. Nearly all bis-acrylamides, bis-*sec*-amines and *prim*-amines available on the market or described in the literature are potential monomers. Consequently, PAAs are a polymer class with high structural versatility. In particular, they can be designed to be biocompatible and degradable [4,5,6]. All PAAs described so far are highly hydrophilic and in most cases water soluble. Many of them have shown potential as complexing agents of heavy metal ion [7], constituents of sensors [8,9,10], heparin complexing agents [11,12,13], drug carriers [14,15,16], antiviral drugs [17,18] and transfection promoters [19,20,21,22]. Proteins graft copolymers have been also described [23].

Cross-linked PAAs are obviously insoluble but swell in water giving hydrogels. Some of these hydrogels proved good scaffolds [24,25,26] for cell culture and promoted cell adhesion and proliferation both in vitro and in vivo. In the form of tubes, they acted as excellent resorbable scaffolds for sciatic nerve regeneration in rats [24]. Undoubtedly, these properties were mediated by their positive charge at the physiological pH which, however, was too low to elicit cellular toxicity. PAA hydrogels showed poor mechanical properties when swollen in water and were hard and brittle when dry. This has hampered their widespread use as implantable biomaterials. To overcome this problem, several approaches have been investigated, such as the preparation of PAA nanocomposite hydrogels with biocompatible nanosized inorganic fillers, such as montmorillonite [27], or their reinforcement with nanofibrous PLLA (poly(L-lactic acid)) mats previously aminated by plasma treatment with nitrogen [28]. In the latter case, the hydrogels were synthesized by embedding the PLLA mat in the curing solution. The amino groups present on the PLLA fibers participated in the polyaddition reaction and gave rise to matrix-fiber covalent bonds, which ensured the stability of the composite in water. Both approaches were only partially successful. Inorganic nanofillers greatly improved the overall mechanical properties of PAA hydrogels but failed to make them susceptible to being attached to body tissues by stitching. As for the PLLA reinforced composite hydrogels, their preparation was difficult to scale up. So far, no attempts have been made to obtain PAAs inherently endowed with potential as structural materials. This paper aims to fill this gap by reporting the synthesis and properties of a small library of hitherto undescribed semi-crystalline hydrophobic PAAs (H-PAAs) that show promise in this regard.

## 2. Materials and Methods

**Materials**. Acryloyl chloride (97%), sodium hydroxide (>98%), 1,6-hexanediamine (98%), 1,8-octanediamine (98%), 1,10-decanediamine (98%), 1,12-dodecanediamine (≥97%), 2,6-di-*tert*-butyl-*p*-cresol (≥99%), *N,N*’-dimethylethylenediamine (DM2) (99%), *N,N’*-dimethyl-1,6-hexanediamine (DM6) (97%), *N,N’*-diethylethylenediamine (DE2) (95%), *N,N’*-dibenzylethylenediamine (DB2) (97%), piperazine (PIP) (99%), benzyl alcohol (≥99.8%), alcohol-free chloroform (≥99.8%), diethyl ether (≥99%), CDCl_3_ (99.8%), CD_3_OD (99.96%), D_2_O (99.9%) and CD_3_COOD (99.9%) were purchased from Sigma-Aldrich (Milano, Italy) and used as received.

**Synthetic procedures**. *N,N’*-*Dodecamethylene-bis-acrylamide (B12)*. In a 1 L four-necked round bottom flask, equipped with a thermometer, a mechanical stirrer and two dropping funnels, 1,12-dodecanediamine (31.0 g, 150.0 mmol) was suspended in CHCl_3_ (800 mL). Under vigorous stirring, the suspension was cooled to 0 ± 1 °C with an acetone/ice bath. Acryloyl chloride (27.6 mL, 329.5 mmol) in CHCl_3_ (18 mL) and NaOH (13.8 g, 338.1 mmol) in H_2_O (34 mL) solutions were added dropwise, maintaining the temperature at around 0–2 °C. At the end of the addition, the reaction mixture was allowed to warm up to room temperature and stirred for 24 h. The solid formed was filtered, washed with an acetic acid/H_2_O 1:2 *v/v* solution (270 mL), then with H_2_O until neutral pH. The resulting product was an off-white solid, which was dried under a vacuum to constant weight (Fraction “a”). Yield: 24.9 g (54.0%). An additional crop was recovered by evaporating the mother liquors to dryness, washing the solid residue with 30% aqueous acetic acid (40 mL) and then water until neutral pH, and drying to constant weight under a vacuum (Fraction “b”). Yield: 2.8 g. The ^1^H-NMR and FT-IR spectra of the two fractions demonstrated that they were identical.

All other bis-acrylamides were prepared following the same procedure adopted for B12. The amounts of amine used and the yields are reported in Table 1.

*Synthesis of B6-DM2 by the solution process. N,N*’-Hexamethylene-bis-acrylamide (11.2 g, 50 mmol), *N,N’*-dimethylethylenediamine (4.5 g, 50 mmol) and benzyl alcohol (46 mL) were introduced into a 100 mL screw cap tube, and the mixture was stirred magnetically at 60 °C for 48 h. The reaction mixture gradually became homogeneous. The solution was finally cooled down to room temperature, precipitated in diethyl ether (500 mL) under stirring and kept at about 5–8 °C overnight. The final product was retrieved by filtering and dried under a vacuum as a whitish solid. Yield 13.3 g (85%). 

All other polymers were prepared as described for B6-DM2 using the amounts of reagents and solvent reported in Table 2. 

*Synthesis of H-PAAs by the bulk process. B12-DM6*. *N,N*’-dimethyl-1,6-hexanediamine (15.4 g, 50 mmol), *N,N’*-dodecamethylene-bis-acrylamide (7.4 g, 32.4 mmol) and 2,6-di-*tert*-butyl-*p*-cresol (150 mg) were introduced into a hermetically sealed cylindrical glass reactor equipped with a thermocouple, a nitrogen inlet and a u-shaped mechanical paddle stirrer. After carefully flushing with nitrogen, the reaction mixture was heated up to 70 °C while homogenizing under stirring. The temperature was then raised up to 140 °C within 1 h and maintained at a constant for further 2 h. After this time, the reacting mixture was allowed to cool down to room temperature. The solidified product was retrieved as a yellowish solid. The yield was quantitative, apart from mechanical losses.

B8-DM6 was prepared as described for B12-DM6, by substituting *N,N’*-octamethylene-bis-acrylamide (12.4 g) for *N,N’*-dodecamethylene-bis-acrylamide and heating up to 150 °C.

**Solubility tests.** H-PAA solubility was determined in a step-by-step procedure. Polymer samples (5 mg) were dispersed in 0.5 mL of solvent and kept under stirring for 15 min. The samples that did not give a clear solution were heated to the solvent boiling point, and the solubility was ascertained. If insoluble, the solutions were diluted with additional solvent (0.5 mL) and the procedure was repeated.

**Nuclear magnetic resonance**. The chemical structure of monomers and polymers was assessed by ^1^H-NMR spectroscopy using a Bruker Avance DPX-400 NMR spectrometer (Milano, Italy) operating at 400.13 MHz. The spectra of bis-acrylamides were collected in CD_3_OD and those of H-PAAs in CDCl_3_ at 25 °C.

**Thermal properties**. Thermogravimetric analyses (TGA) were performed from 35 to 800 °C in both air and nitrogen on a Mettler-Toledo TGA/DSC 2 Star System instrument (Milano, Italy). Samples (5–10 mg) were placed in alumina crucibles and heated at a 10 °C min^−1^ heating rate under a 50 mL min^−1^ nitrogen flow.

Differential scanning calorimetric (DSC) analyses were performed on a Mettler-Toledo DSC823^e^ System (Milano, Italy) as follows: samples (5 mg) were placed in standard aluminum crucibles and heated or cooled at 5 °C min^−1^ under an 80 mL min^−1^ nitrogen flow. The heating program was as follows: 1st step: heating from 25 °C to T_f_; 2nd step: 1 min at T_f_; 3rd step: cooling from T_x_ to 25 °C; 4th step: 1 min at 25 °C; 5th step: heating from 25 °C to T_x_; 6th step: 1 min at T_f_; 7th step: cooling from T_x_ to 25 °C. T_f_ is the maximum temperature typical of each polymer. Result accuracy was ±1 °C and ±0.01 J g^−1^. Before ea ch set of analyses, the instrument was calibrated with an indium standard. T_g_ values were assessed by a TA Waters instrument Model DSCQ20 (Milano, Italy); samples (5.5 mg) were placed in standard aluminum crucibles and heated or cooled at 10 °C min^−1^ under a 80 mL min^−1^ nitrogen flow. The heating program was as follows: 1st step: heating from 25 °C to T_f_; 2nd step: 1 min at T_f_; 3rd step: cooling from T_x_ to −50 °C; 4th step: 1 min at −50 °C; 5th step: heating from −50 °C to T_x_.

**Ignitability**. The ignitability of H-PAAs was assessed by applying a methane flame (20 mm long) directly to the polymer powder (~500 mg) placed on a ceramic-porcelain spoon spatula for 10 s, as already done for other flame retardants [29].

**Preliminary evaluation of resistance to handling and to water contact of H-PAA films**. Films 0.2 Mm thick were obtained by melting polymer samples (100 mg) on a heating plate in a 5 cm^2^ frame at a temperature of 20 °C above their melting point, then pressing with a 1.5 Kg weight for 10 s at the same temperature and finally cooling to room temperature. One half of the films obtained were incubated in water for 1 min, and the handling resistance was compared with that of the corresponding dry films.

**Tensile properties**. Tensile stress-strain tests were performed according to the ISO 37-2011 standard. *b*B12-DM6 and *b*B8-DM6 films 1 mm thick were obtained using a compression molding press (Model C, Carver Laboratory Press, Wabash, IN, USA). The samples (2 g) were first dried at 60 °C overnight in a vacuum oven, then placed between two pre-heated steel plates at the desired temperature (110 °C for B12-DM6 and 120 °C for B8-DM6) and finally subjected to 0.5 ton (191,406 Pa) for 4 min and then to 0.8 ton (306,250 Pa) for 1 min. The samples were then cooled to room temperature using circulating water. From this process, films 0.1 mm thick were obtained. The specimens for the tensile test were obtained using a specific dumbbell (dog-bone like) (total length 70 mm, central zone 25 mm). The samples were conditioned for at least 40 h at 25 °C and 50% relative humidity. Film thickness was measured at three random points using a micrometer, and the result was expressed as an average value. Tensile strength was measured with an INSTROM 5564 dynamometer (Torino, Italy), using a 1 KN load cell and a 2 mm min^−1^ deformation speed. Several replicate measurements (5–10) were performed on each H-PAA sample. 

**Wettability**. Water contact angle measurements were performed with the contact angle system FTA 1000 B CLASS (First Ten Angstroms St. Johns Innovation Center, Cowley Road, Cambridge CB4 OWS, UK) according to the following protocol: deionized water drops of 30 ± 2 μL in volume were cast on a flat portion of H-PAA film. The shape of the drop was recorded with a high-speed framing camera. The contact angle was assessed by measuring the angle formed between the substrate surface and the tangent drawn from the edge of the drop. Measurements were taken after a static time of 30 s. Contact angles were measured at two points, i.e., near the center and near the edge of the specimens.

## 3. Results and Discussion

As stated in the introduction, the aim of this work was to prepare linear semi-crystalline hydrophobic PAAs (H-PAAs) endowed with potential as structural materials in terms of processability and fair mechanical properties. For this purpose, a small library of H-PAAs was prepared from the aza-Michael polyaddition of four α,ω-bis-acrylamides obtained from linear aliphatic C6-C12 bis-*prim*-amines and several bis-*sec*-amines, including linear α,ω-aliphatic N-substituted bis-*sec*-amines; a cyclic bis-*sec*-amine, namely piperazine; and an α,ω-arylalkyl N-substituted bis-*sec*-amine, namely *N,N*’-dibenzylethylenediamine. Regardless of the aliphatic portion, *prim*-amines were not considered in this study, as preliminary experiments invariably resulted in amorphous and sticky polymers. 

### 3.1. Synthesis of Hydrophobic Bis-Acrylamides

**Scheme 1:**
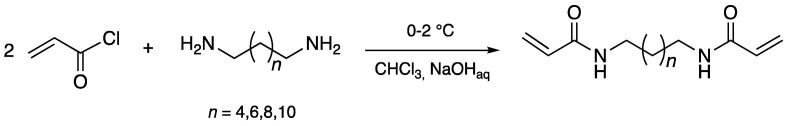
Synthesis of the bis-acrylamides used in H-PAA syntheses.

All bis-acrylamides used, i.e., *N,N’*-dodecamethylene-bis-acrylamide (B12), *N,N*’-decamethylene-bis-acrylamide (B10), *N,N’*-octamethylene-bis-acrylamide (B8) and *N,N’*-hexamethylene-bis-acrylamide (B6) (Table 1), were synthesized by the Schotten-Baumann reaction [30,31,32]. A solution of acryloyl chloride in alcohol-free chloroform and an aqueous sodium hydroxide solution were added dropwise with vigorous stirring to a solution or fine suspension of the diamine in chloroform (Scheme 1). The reaction mixture was kept below 5 °C under stirring throughout the reaction process. The final product, precipitated as a solid and after filtration, was repeatedly washed with dilute acetic acid and then water to remove any traces of unreacted diamine. The chloroform mother liquors were first dried with anhydrous sodium sulphate and then evaporated under a vacuum. A second crop of product was recovered, and the residue was washed with dilute acetic acid and then water. Yields ranged from 60 to 70%. 

**Figure 1:**
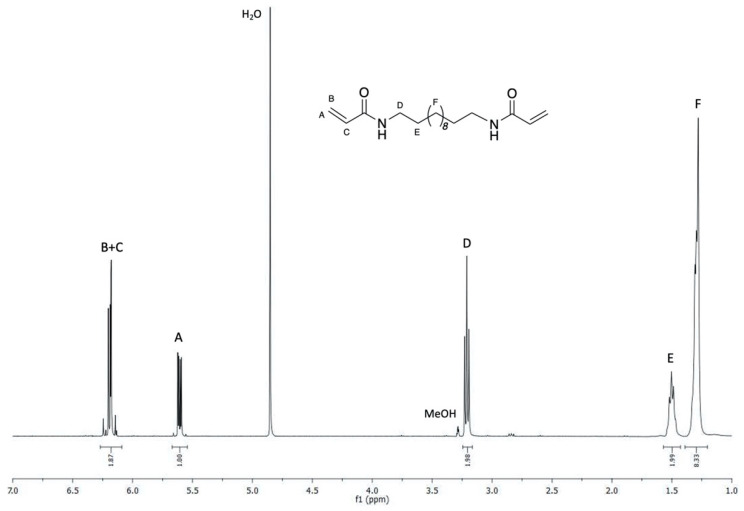
^1^H-NMR spectrum of bis-acrylamide B12 obtained in CD_3_OD using a 400 MHz Avance Brüker spectrometer. Water assignment was in agreement with the literature [33,34].

The structures of all bis-acrylamides were confirmed by ^1^H-NMR spectroscopy. As an example, the ^1^H-NMR spectrum of B12, performed on CD_3_OD, is reported in Figure 1, while the spectra of all other bis-acrylamides are reported in Figures S1–S3. It may be observed that no separate signals for N-H amidic protons can be identified in the ^1^H-NMR spectrum of B12 reported in Figure 1. This may be due to different reasons, either rapid proton-deuterium exchange with the relatively acid O-D group of CD_3_OD or, as frequently observed, the N-H resonance peak may be very broad and hidden by overlapping with other signals. 

### 3.2. Synthesis of H-PAAs by the Solution Process

**Scheme 2:**
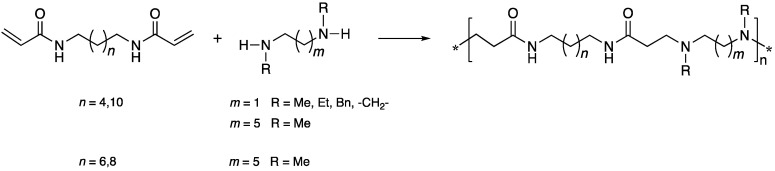
Synthesis of H-PAAs.

Twelve not previously described H-PAAs (Table 3) were obtained first by solution polymerization, which is the common synthetic procedure of PAAs reported so far, by combining the bis-acrylamides B6, B8, B10 and B12 (Table 2, Section 2. Materials and Methods) with the five bis-*sec*-amines reported in Scheme 2.

Specifically, B12 and B6 were combined with all five bis-*sec*-amines, while, for comparison purposes, B10 and B8 were only combined with *N,N*’-dimethyl-1,6-hexanediamine (DM6). The aza-Michael polyaddition requires solvents with mobile hydrogens, such as water and alcohols, which in principle are poor solvents for hydrophobic polymers. Benzyl alcohol has been shown to be a good reaction medium for both precursor monomers and H-PAAs. All solution polymerization experiments were conducted at 60 °C. The resulting polymers were finally isolated by precipitating with ether. This process required large volumes of solvents, longer reaction times and tedious procedures to remove benzyl alcohol.

All H-PAAs were characterized by ^1^H-NMR and FT-IR/ATR spectroscopies. As an example, the ^1^H-NMR spectrum of B12-DM2 performed on CDCl_3_ is reported in Figure 2, while the ^1^H-NMR spectra of all other H-PAAs are reported in Figures S4–S14. The FT-IR spectra of all H-PAAs are reported in Figures S17–S27.

All characterizations were in agreement with the proposed structures, apart from the fact that all ^1^H-NMR spectra revealed the presence of a benzyl alcohol residue (1–3% *w*/*w*). This was taken into account in the interpretation of the TGA analyses (see below). The ^1^H-NMR spectra allowed us to estimate the number average molecular weights, ${\bar{M}}_{n}$, of the H-PAAs (see Table 2, Section 2. Materials and Methods) from the ratio between the integrals of the resonance peaks relative to the terminal units and the integrals of the resonance peaks relative to the internal repeating units. Size exclusion chromatography proved inadequate for determining the molecular weights of H-PAAs because they were strongly retained by the stationary phase, regardless of the mobile phases used. It can be seen that the ^1^H-NMR spectra of B12-DB2 (Figure S5) and B6-DB2 (Figure S12), both derived from *N,N*’-dibenzylethylenediamine, showed significant amounts of residual double bonds, and their ${\bar{M}}_{n}$ values were less than 3000. Furthermore, even considering that these polymers were soft and sticky, they were not further considered. 

Regarding solubility properties, it can be observed that, apart from the piperazine-based samples B12-PIP and B6-PIP, all H-PAAs had similar solubility properties (Table 4). They were completely soluble in chlorinated solvents and boiling alcohols but insoluble in ethers, acetone, dimethyl sulfoxide, water and 30% aqueous acetic acid. In contrast, both B12-PIP and B6-PIP were insoluble in chlorinated solvents and boiling alcohols but soluble in dilute acetic acid. 

### 3.3. Synthesis of H-PAAs by the Bulk Process

A preliminary screening (see below) resulted in the selection of B12-DM6 and B8-DM6 as the best-performing H-PAAs, which were successfully synthesized even following a bulk process. Mixtures of equimolar monomers, added with a radical polymerization inhibitor, were gradually heated under stirring in nitrogen from 70 to 140 °C and then kept at the latter temperature for 2 h. The reaction mixture was then cooled down and the product used as retrieved. The resulting products, named *b*B12-DM6 and *b*B8-DM6, were characterized by ^1^H-NMR spectroscopy (Figures S15 and S16). Aside from the obvious absence of solvent impurities, their spectra were nearly identical to those of B12-DM6 and B8-DM6. The ${\bar{M}}_{n}$ values of *b*B12-DM6 and *b*B8-DM6 were of the same order as those of B12-DM6 and B8-DM6. Advantages of the bulk process were that it was much faster than the solution process and avoided the presence of residual solvent. To the best of our knowledge, no examples of PAA synthesis by a bulk polymerization process have been reported so far. 

### 3.4. Thermal Stability of H-PAAs

**Figure 3:**
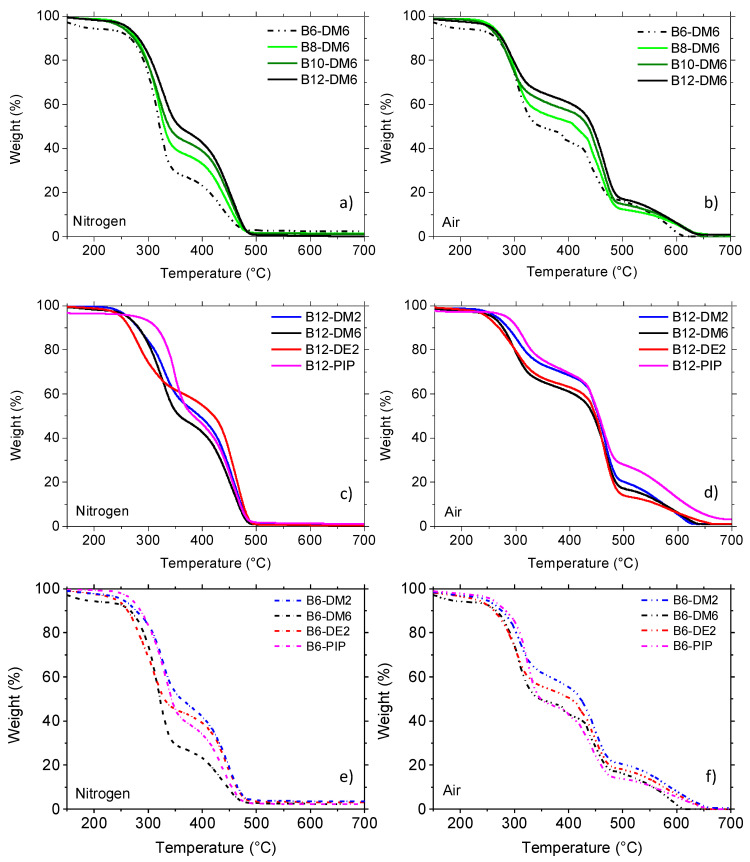
TGA thermograms of H-PAAs: panels (**a**,**c**,**e**) in nitrogen; panels (**b**,**d**,**f**) in air.

Figure 3 shows the TGA thermograms of H-PAAs conducted on both nitrogen and air in the temperature range of 150–700 °C. Table 5 reports the onset decomposition temperature at 10% weight loss, T_onset10%_; the temperatures at maximum weight loss rate, T_max1,_ T_max2_ and T_max3_; and the residual mass fractions measured at 400 and 550 °C, RMF_400_ and RMF_550_. Consistent with what was previously reported for hydrophilic PAAs studied as flame retardants for cotton [29], H-PAAs showed complex multimodal weight-loss curves in both nitrogen and air, deriving from a multistep degradation mechanism. All considered, H-PAAs were characterized by fairly high thermal stability in both atmospheres—higher overall than those of linear water-soluble PAAs—as evidenced by the T_onset10%_ and T_max_ values. In particular, the T_onset10%_ values of all the considered H-PAAs were >265 °C in nitrogen and >266 °C in air, while the T_max1_ values were 280 °C in nitrogen and 290 °C in air.

In nitrogen, the T_onset10%_ values of H-PAAs derived from the same bis-acrylamide (B12 or B6) decreased by varying the structure of bis-*sec*-amine in the order PIP > DE2 > DM2 > DM6. Furthermore, all H-PAAs, regardless of composition, underwent two main decomposition steps in the ranges of 250–350 °C and 350–500 °C. The RMF_400_ values were in all cases relevant. In particular, as regards H-PAAs derived from the same bis-*sec*-amine, i.e., DM6, and different bis-acrylamides, it was found that the longer the bis-acrylamide hydrocarbon chain, the higher the RMF_400_ value, i.e., 43, 39, 33 e 23% for B12-DM6, B10-DM6, B8-DM6 and B6-DM6, respectively (Figure 3a). Regarding the dependence of RMF_400_ on the structure of the bis-*sec*-amine moieties in combination with the same bis-acrylamide, no clear correlation was observed (Figure 3c,e). Negligible (<1%) RMF was observed for all H-PAAs from 500 °C and above. 

In air, both T_onset10%_ and T_max2_ values did not differ significantly from those observed in nitrogen, whereas the T_max1_ values were 2–32 °C lower than in nitrogen, with the sole exception of B12-DE2, whose T_max1_ was 10 °C higher. Furthermore, in the range of 300–450 °C, all H-PAAs showed a lower weight loss rate in air than in nitrogen and, as a result, the RMF_400_ values were remarkably higher. This has been attributed to the phenomenon of intumescence, which occurs by heating the air and is recognized as a characteristic of PAAs [29]. In air, as in nitrogen, the RMF_400_ values of H-PAA derived from DM6 increased with the increase in the bis-acrylamide hydrocarbon chain length, i.e., they were 61, 57, 52 and 43% for B12-DM6, B10-DM6, B8-DM6 and B6-DM6 (Figure 3b). No clear correlation between RMF_400_ values and the structure of the amine moiety was found (Figure 3d,f). In addition to the decomposition steps observed in nitrogen in the temperature ranges of 250–350 °C and 350–500 °C, a third decomposition step was observed in the range of 500–650 °C. RMF_550_ values were between 10 and 13%, while in nitrogen they were less than 1%, and almost vanished above 600–650 °C. 

The thermal data of *b*B12-DM6 and *b*B8-DM6 in air and nitrogen, prepared by the bulk process, were almost superimposable to those of samples obtained from the solution process (Figure S30 for B12-DM6). 

Comparing the thermal data of H-PAAs with those of common commercial polymers, it was observed that the T_onset10%_ and T_max_ of H-PAAs did not differ significantly when switching from air to nitrogen, while those of PP [35] and HDPE [35,36] were about 50–150 °C lower in air than in nitrogen. Furthermore, above T_max_, the RMF of PE and PP in the air drops to a negligible amount. In contrast, in air H-PAAs showed a substantial RMF (15–30%) up to 500–650 °C, where a third T_max_ occurred. It may be observed that the T_onset10%_ values of polybutylene terephthalate (PBT) [37], polyethylene terephthalate (PET) [38,39] and nylon 6 (PA6) [40,41] were higher than those of H-PAAs. Furthermore, they showed a single T_max_ value, while H-PAAs showed two T_max_ values, the first lower than- and the second, comparable to those of PBT, PET and PA6. Furthermore, the RMF values of PBT, PET and PA6 above T_max_ are significant (18% at 480 °C for PET [38] 8% at 500 °C for PA6 [40]) but invariably lower than those of H-PAAs, which become negligible only above 600–650 °C. 

**Figure 4:**
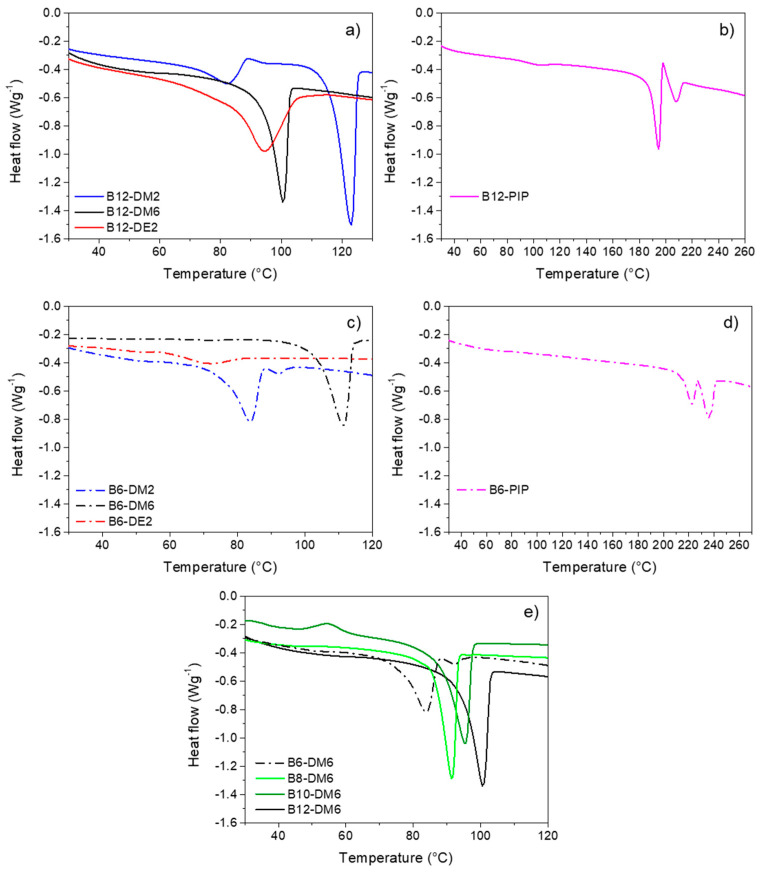
Differential scanning calorimetric (DSC) thermograms pertaining to the 2nd heating cycle at 10 °C min^−1^ of: (**a**) B12-DM2, B12-DM6 and B12-DE2; (**b**) B12-PIP; (**c**) B6-DM2, B6-DM6 and B6-DE2; (**d**) B6-PIP; (**e**) B6-DM6, B8-DM6, B10-DM6 and B12-DM6.

Figure 4 shows the DSC thermograms of H-PAAs relating to the 2nd heating cycle at 10 °C min^−1^, and Figure S31 shows those pertaining to the cooling cycle. The onset melting temperatures, T_onset_; the melting and crystallization temperatures, T_m_ and T_c_; the melting and crystallization enthalpies, ΔH_m_ and ΔH_c_; and the glass transition temperatures, T_g_, are listed in Table 6. All H-PAAs, with the exception of B6-DE2, were semi-crystalline and provided evidence of melting (Figure 4c) and crystallization (Figure S31c). In particular, samples B12-PIP, B6-PIP, B12-DM2 and B6-DM6 showed bimodal melting traces. Quite surprisingly, B10-DM6 showed evidence of melting (Figure 4e) but not of extensive recrystallization (Figure S31e). Furthermore, both PIP-based samples exhibited particularly high T_m2_ values: 196/216 and 223/236 °C for B12-PIP and B6-PIP, respectively (Table 6 and Figure 4b,d).

H-PAAs derived from both B12 (Figure 4a) and B6 (Figure 4c) showed the same ranking of T_m_ values based on the structure of the amine moiety: PIP > DM2 > DM6 > DE2. It should be remembered that B6-DE2 did not melt nor crystallize. Within the DM6 series, T_m2_ values (100, 95, 90 and 84 °C for B12-DM6, B10-DM6, B8-DM6 e B6-DM6, respectively) were clearly dependent on the bis-acrylamide chain length and decreased by decreasing the number of the bis-acrylamide carbon atoms (Figure 4e). The ΔH_m2_ values also decreased by decreasing the number of carbon atoms in the series (63.3, 62.0, 57.1 and 51.1 Jg^−1^ for B12-DM6, B10-DM6, B8-DM6 e B6-DM6, respectively). The differences between the thermal data of B12-DM6 and *b*B12-DM6, and between those of B8-DM6 and *b*B8-DM6, were not relevant (Figure S32).

Regarding the cooling cycle, both B12-PIP and B6-PIP showed the highest crystallization temperatures, T_c_, of the B12- and B6-series (179 °C for B12-PIP and 200 °C for B6-PIP), as already observed for T_m2_ values. Furthermore, within the DM6-series, T_c_ showed almost the same trend observed for T_m2_, with the exception of B12-DM6 and B12-DE2. The significant increase in the melting enthalpy of B12-DM6 compared with B8-DM6 can be explained by the greater mobility of the B12 chains that can arrange in larger crystalline domains. 

The glass transition temperatures, T_g_, of H-PAAs were between −10 and −23 °C, except for B12-PIP and B6-PIP, whose T_g_ values were 32 °C and 55 °C, respectively. Remarkably, the T_g_ of B12-DE2 was not detectable. Within the DM6 series, the T_g_ values decreased slightly as the bis-acrylamide chain length increased (−19, −19, −20, and −23 °C for B12-DM6, B10-DM6, B8-DM6 and B6-DM6, respectively). The T_g_ values of *b*B12-DM6 and *b*B8-DM6 were slightly higher than those of the corresponding samples obtained in solution. This is likely due to the presence, in the latter, of the residual impurities of benzyl alcohol. 

### 3.5. Preliminary Evaluation of the Effect of Water Contact on the Macroscopic Properties of H-PAA Films

H-PAA films with an average thickness of 0.2 mm were prepared by compression molding. The specimens were visually evaluated for their flexibility and ability to withstand small stresses and then placed in contact with water to ascertain the effect of water on the observed properties (see Table 7). These tests provided the criteria for selecting B12-DM6 and B8-DM6 as the best H-PAA samples for studying the feasibility of the bulk polymerization process and further evaluating the wettability and tensile properties of the resulting *b*B12-DM6 and *b*B8-DM6 samples. 

### 3.6. Ignitability Tests

**Figure 5:**
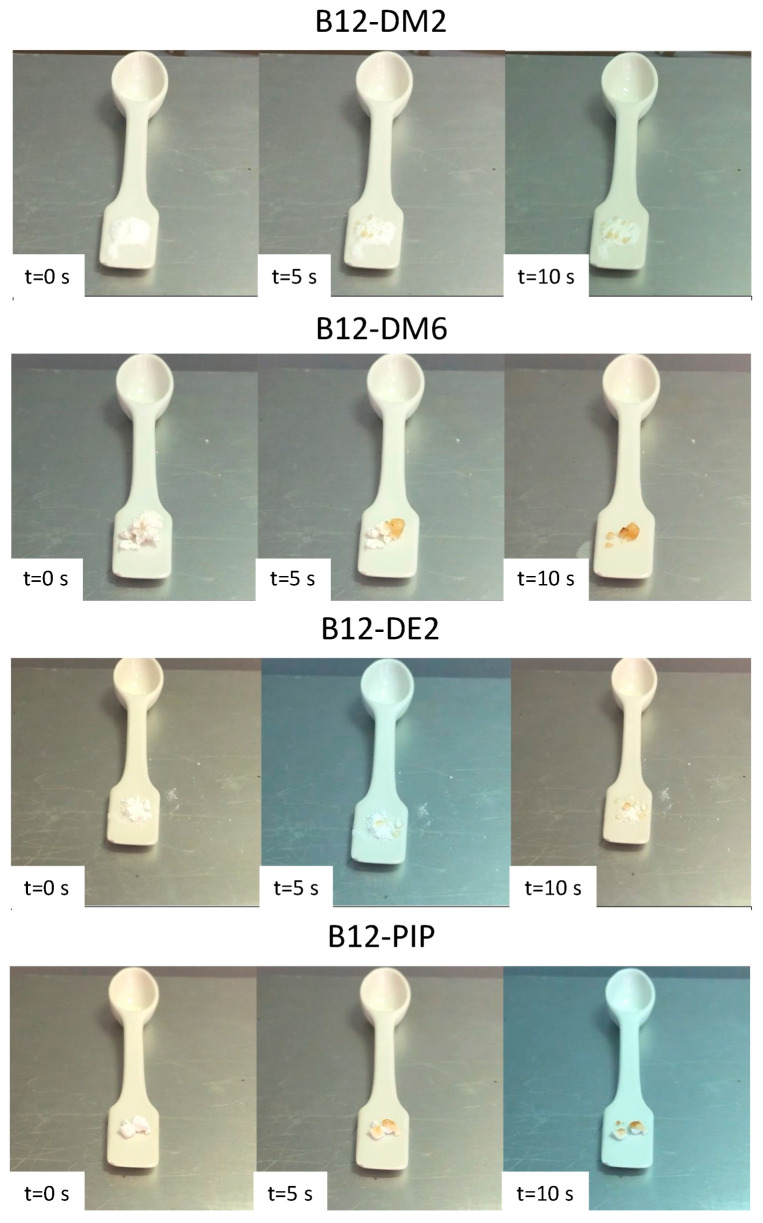
Snapshot of the results of ignitability tests carried out on H-PAA powders.

The ignitability of H-PAAs was assessed by direct flame impingement for 10 s on powdered samples (snapshots reported in Figure 5). The samples did not ignite when the flame was applied and exhibited minimal weight loss (<5%). Their surface melted and decomposed, yellowing slightly, and produced carbonaceous crusts that protected the underlying powders, which remained white and apparently intact. This behavior testified the tendency of H-PAAs to intumesce upon heating and was in line with previous ignitability data obtained with hydrophilic PAAs [29,42]. It also demonstrated that inserting a large hydrophobic moiety in their repeating unit did not significantly affect their flame resistance. The inherent intumescent behavior of PAAs is a rarely seen feature in polymeric flame retardants. Similar behavior, in fact, has been observed only in DNA and in some proteins for which, however, industrial exploitation for this purpose is almost inconceivable [43,44,45].

### 3.7. Preliminary Evaluation of Tensile Properties of bB12-DM6 and bB8-DM6

**Figure 6:**
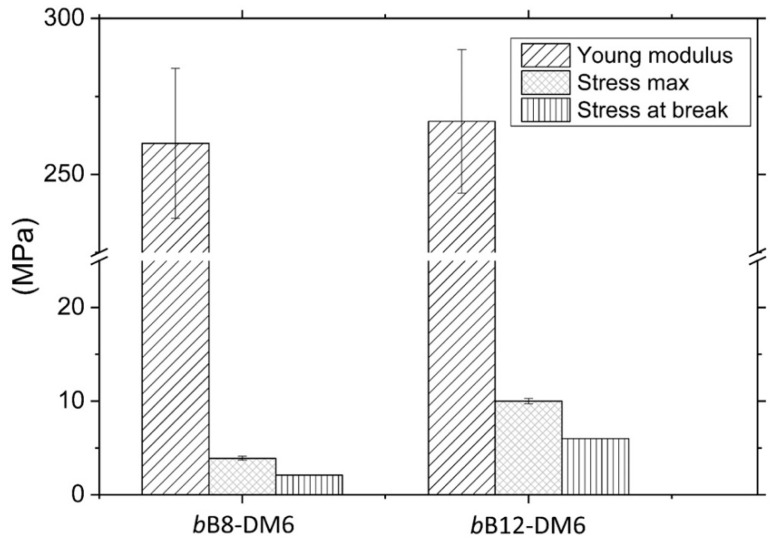
Graphical representation of *b*B12-DM6 and *b*B8-DM6 tensile properties.

The tensile properties of *b*B12-DM6 and *b*B8-DM6 were investigated on 0.1 mm thick dog bone shaped specimens following the ISO 37-2011 standard. The results are shown in Figure 6 and Table 8.

It is evident that *b*B12-DM6 and *b*B8-DM6 have a similar Young’s modulus. It should be noted that they have similar molecular weights, despite their degrees of polymerization being somewhat different. Furthermore, the samples did not undergo a visible yield before failure. It can also be observed that all tensile data, i.e., the strain at break, the stress at break and the maximum stress of the two samples, differed significantly. Not surprisingly, these values increased from *b*B8-DM6 to *b*B12-DM6, i.e., by increasing the length of the bis-acrylamide hydrocarbon chain. The greater maximum stress value of B12-DM6, compared with B8-DM6, is linked to the greater crystallinity of the first polymer, as also highlighted by the thermal data.

### 3.8. Wettability

**Figure 7:**
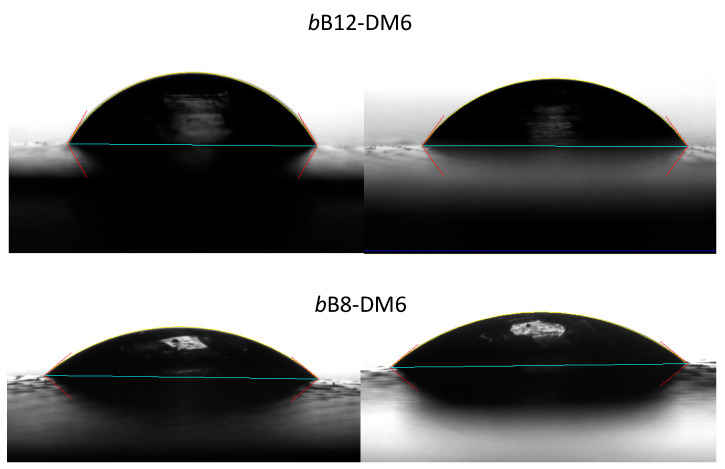
Contact angle measurements on *b*B12-DM6 (**upper** row) and *b*B8-DM6 (**lower** row) films: contact point close to the border (**left** panels); contact point near the center of the specimen (**right** panels).

The wettability of *b*B12-DM6 and *b*B8-DM6 was assessed by measuring the contact angle with water using the sessile drop method. Contact angles were measured both near the center and at the edge of the samples. Results are reported in Figure 7 and Table 9. It is evident that the wettability of *b*B12-DM6 was significantly lower than that of *b*B8-DM6, as expected. In fact, the proportion between the hydrophobic chains and the hydrophilic amide and amine groups of the former is higher. 

## 4. Conclusions

The aim of this work was to prepare linear hydrophobic semi-crystalline PAAs with fair physical and mechanical properties using hydrophobic bis-acrylamides and bis-sec-amines as monomers. A small library of twelve linear H-PAAs was synthesized and characterized. A preliminary investigation into the relationship between structure and relevant physical properties allowed us to select a limited number of samples, investigated with regards to wettability and tensile properties.

H-PAAs were prepared by both solution and bulk processes. Initially, all the samples were prepared by a solution process, by analogy with the linear PAAs described so far in the literature, employing benzyl alcohol as the solvent. The samples selected for wettability and tensile tests, i.e., *b*B12-DM6 and *b*B8-DM6, were also prepared by a bulk process, never previously described for the synthesis of PAAs. The bulk process was carried out under a nitrogen atmosphere at a temperature well above the melting points of both the monomers and the resulting polymers and in the presence of a radical inhibitor. This process proved more effective as it led to samples with no need for further purification. The number of the average molecular weights of *b*B12-DM6 and *b*B8-DM6 did not differ significantly from those of the corresponding samples obtained from the solution process.

The H-PAAs obtained by the solution process were first screened for the molecular weight obtainable and, subsequently, physical properties including thermal properties, filmability, flammability, apparent strength and water resistance. Samples B12-DB2 and B6-DB2 were discarded due to their low molecular weight, as revealed by the significant presence of residual double bonds in their ^1^H-NMR spectra. Furthermore, they were sticky and apparently unable to crystallize. All remaining H-PAAs were semi-crystalline, with the sole exception of B6-DE2. The melting temperatures of the H-PAAs derived from the same amine, namely DM6, clearly increased by increasing the length of the bis-acrylamide moiety. Those of the H-PAAs derived from the same bis-acrylamide and different bis-amines also depended on the structure of the amine substituent. The order for H-PAAs-derived B12 and B6 was PIP >> DM2 > DM6 > DE2. B6-DE2 did not crystallize.

Overall, the thermal stability of H-PAAs was considerably higher, in terms of T_onset10%_ and T_max_, than those of the hydrophilic PAAs studied so far [29]. Furthermore, as hydrophilic PAAs, they were significantly more stable in air than in nitrogen, and they exhibited greater residue formation, which was not present in the TGA traces of many commercial polymers, including Nylons and PET. The residual mass fraction of H-PAAs in the air at 550 °C varied between 10 and 13%. The residual mass fractions at 400 °C made it possible to identify the correlations between thermal stability and the number of carbon atoms in the bis-acrylamide moiety. The influence of the amino moiety structure was not uniquely identified, except in the case of the H-PAAs derived from piperazine, which were the most thermally stable considering both T_onset10%_ and T_max_.

Ignitability tests proved that resistance to direct flame impingement was excellent in all cases. The samples melted but did not burn and partially decomposed on the surface, turning yellow. The interior remained apparently intact.

H-PAAs were completely insoluble in water. However, the films of half of them became brittle when in contact with water. The H-PAAs most resistant to contact with water were B12-DM6 and B8-DM6. Subsequently, they were synthesized in bulk and characterized by wettability and tensile tests. They showed a similar Young’s modulus (260 for B8-DM6 and 267 MPa for B12-DM6), while the maximum stress, stress at break and strain at break increased from B8-DM6 to B12-DM6, apparently depending on the number of methylene groups in the bis-acrylamide moieties.

## Figures and Tables

**Figure 2:**
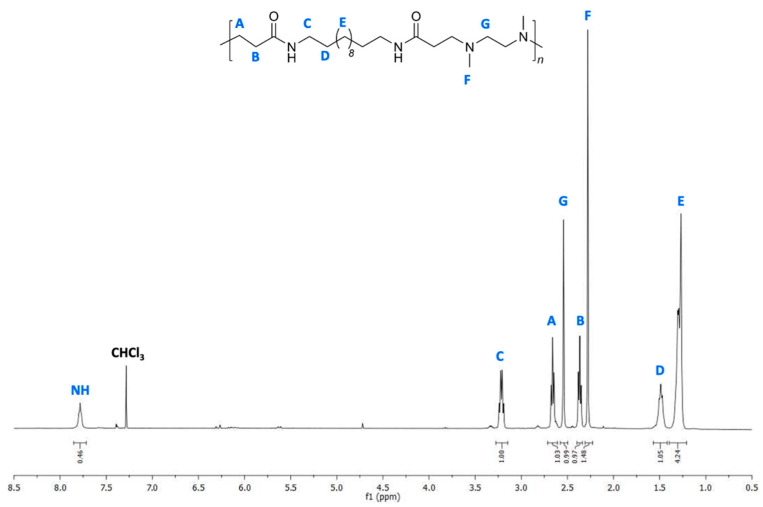
^1^H-NMR spectrum of H-PAA B12-DM2 obtained in CDCl_3_ using a 400 MHz Avance Brüker spectrometer.

**Table 1:** Amounts of reagents used in the synthesis of bis-acrylamides ^(a)^.

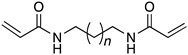				
Code	N ^(b)^	Amine ^(c)^ (g)	Yield (g)	Yield (%)
B12	10	31.0	27.7	60.0
B10	8	26.4	24.0	57.0
B8	6	22.1	26.5	70.0
B6	4	17.8	20.5	61.0

^(a)^ In all cases, the amount of acryloyl chloride was 29.9 g (330 mmol). ^(b)^ Number of CH_2_ groups reported in parentheses; ^(c)^ 150 mmol.

**Table 2:** Amounts of reagents used in the synthesis of H-PAAs by solution ^(a,b)^ and bulk processes. ^(b,c)^

Sample Code ^(d)^	Bis-Acrylamide (g)	Bis-Amine (g)	Benzyl Alcohol (mL)	Time (h)	Yield [g, (%)]	${\bar{M}}_{n}$
B12-DM2	15.4	4.5	57.0	48	17.7 (89)	9700
B12-DM6	15.4	7.4	65.0	48	21.3 (93)	>10,000
B12-DE2	15.4	5.9	61.0	48	18.7 (88)	6000
B12-DB2	15.4	12.4	83.0	96	19.5 (70)	<3000
B12-PIP	15.4	4.4	59.0	48	19.6 (99)	6400
B10-DM6	14.0	7.4	61.0	48	20.4 (95)	6000
B8-DM6	12.6	7.4	60.0	48	18.5 (92)	6400
B6-DM2	11.2	4.5	46.0	48	13.3 (85)	>10,000
B6-DM6	11.2	7.4	55.0	48	15.5 (83)	>10,000
B6-DE2	11.2	5.9	51.0	48	11.3 (66)	6000
B6-DB2	11.2	12.4	69.0	96	n.d. ^(e)^	<3000
B6-PIP	11.2	4.4	45.0	48	(98)	>10,000
*b*B12-DM6 ^(f)^	15.4	7.4	-	3	(~100)	7500
*b*B8-DM6 ^(f)^	12.6	7.4	-	3	(~100)	6400

^(a)^ Temperature = 60 °C. ^(b)^ Monomer amounts: 50 mmol. ^(c)^ Temperature = 70–140 °C. ^(d)^ B12: 1,12-dodecamethylene-bis-acrylamide; B10: 1,10-decamethylene-bis-acrylamide; B8: 1,8-octamethylene-bis-acrylamide; B6: 1,6-hexamethylene-bis-acrylamide; DM2: *N,N*’-dimethylethylenediamine; DM6: *N,N’*-dimethyl-1,6-hexanediamine; DE2: *N,N’*-diethylethylenediamine; DB2: *N,N’*-dibenzylethylenediamine; PIP: piperazine. ^(e)^ n.d.: not determined. ^(f)^ Sample obtained by the bulk process.

**Table 3:** Structure of H-PAAs.

Polymer Code	Structure of the Repeat Unit
B12-DM2	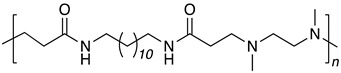
B12-DE2	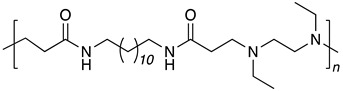
B12-DB2	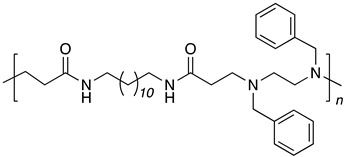
B12-DM6	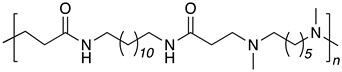
B12-PIP	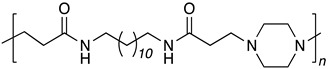
B10-DM6	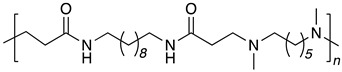
B8-DM6	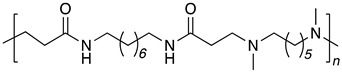
B6-DM2	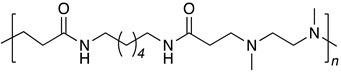
B6-DE2	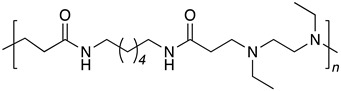
B6-DB2	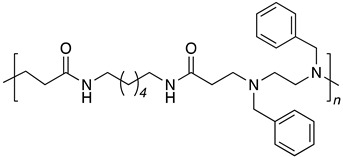
B6-DM6	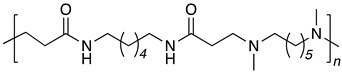
B6-PIP	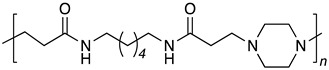

**Table 4:** Solubility of H-PAAs ^(a)^.

	B12-DM2	B12-DM6	B12-DE2	B12-DB2	B12-PIP	B10-DM6	B8-DM6	B6-DM2	B6-DM6	B6-DE2	B6-DB2	B6-PIP
Methanol	i	i	sh	sh	i	sh	sh	sh	sh	sh	sh	i
Ethanol	sh	sh	sh	sh	i	sh	sh	sh	sh	sh	sh	i
Isopropanol	sh	sh	sh	sh	i	sh	sh	sh	sh	sh	sh	i
Butanol	sh	sh	sh	sh	sh	sh	sh	sh	sh	sh	sh	i
Benzyl alcohol	sh	s	s	s	sh	sh	sh	sh	s	S	s	sh
Acetone	i	i	i	i	i	i	i	i	i	i	i	i
Dimethyl sulfoxide	i	i	i	i	i	i	i	i	i	i	i	i
Diisopropyl ether	i	i	i	i	i	i	i	i	i	i	i	i
Diethyl ether	i	i	i	i	i	i	i	i	i	i	i	i
Chloroform	s	s	s	s	i	s	s	s	s	s	s	i
Dichloromethane	i	s	s	s	i	s	s	s	s	s	s	i
Acetic acid/ water 1:2	i	i	i	i	s	i	i	i	i	i	i	s
Water	i	i	i	i	i	i	i	i	i	i	i	i

^(a)^ i: insoluble; s: soluble; sh: soluble at boiling point.

**Table 5:** Thermal data collected by thermogravimetric analyses (TGA).

Polymer	Nitrogen				Air					
	T_onset10%_^(a)^ (°C)	T_max1_^(b)^ (°C)	T_max2_ (°C)	RMF_400_^(c)^ (%)	T_onset10%_ (°C)	T_max1_ (°C)	T_max2_ (°C)	T_max3_ (°C)	RMF_400_ (%)	RMF_550_^(d)^ (%)
B12-DM2	281	330	460	49	285	305	462	610	68	14
B12-DE2	265	280	462	55	268	290	464	575	63	11
B12-DM6	270	323	459	43	269	299	465	610	61	13
B12-PIP	315	347	458	46	297	315	460	585	69	22
B10-DM6	273	320	456	39	270	292	465	620	57	12
B8-DM6	278	320	452	33	278	300	442/460	610	52	11
B6-DM2	278	325	445	42	275	310	448	581	55	16
B6-DE2	265	304	445	39	268	302	447	580	50	12
B6-DM6	266	316	440	23	266	305	450	587	43	10
B6-PIP	286	330	438	34	282	324	446	575	43	11
*b*B12-DM6 ^(^^e)^	271	318	457	41	281	300	464	617	61	13
*b*B8-DM6 ^(^^e)^	279	322	450	34	277	300	441/462	612	53	10

^(a)^ T_onset10%:_ onset decomposition temperature at 10% weight loss; ^(b)^ T_max_: temperature at maximum weight loss rate; ^(c)^ RMF_400_ residual mass fractions measured at 400 °C; ^(d)^ RMF_550_ residual mass fractions measured at 550 °C; ^(e)^ sample produced by bulk polymerization process. RMF_550_ in air is negligible for all samples.

**Table 6:** Thermal data collected by DSC.

Polymer	1st Heating Cycle			Cooling Cycle			2nd Heating Cycle			
	T_onset,m1_^(a)^ (°C)	T_m1_^(b)^ (°C)	ΔH_m1_^(c)^ (Jg^−1^)	T_onset,cr_^(d)^ (°C)	T_cr_^(e)^ (°C)	ΔH_cr_^(f)^ (Jg^−1^)	T_onset,m2_^(g)^ (°C)	T_m2_^(h)^ (°C)	ΔH_m2_^(i)^ (Jg^−1^)	T_g_^(l)^ (°C)
B12-DM2	70	83	−23.4	97	91	49.3	116	123	−76.6	−10
B12-DM6	-	-	-	71	61	58.6	95	100	−63.3	−19
B12-DE2	-	-	-	80	74	82.3	83	95	−95.5	n.d. ^(m)^
B12-PIP	188	195	−51.3	181	179	48.5	198	196/216	−18.3	+32
B10-DM6	-	-	-	53	44	3.2	88	95	−62.0	−19
B8-DM6	-	-		53	46	54.4	86	90	−57.1	−20
B6-DM2	-	-	-	78	72	53.6	103	111	−54.5	−13
B6-DM6	76	84	−37.1	55	50	48.1	89	84	−51.1	−23
B6-DE2	-	-	-	-	-	-	-	-	-	-
B6-PIP	216	222	−13.2	205	200	53.4	228	223/236	−24.2	+55
*b*B12-DM6	-	-	-	68	62	34.6	98	105	−72.2	−10
*b*B8-DM6	-	-	-	63	57	53.0	93	100	−50.8	−16

1st Heating cycle: ^(^^a)^ T_onset, m1_: the onset temperature, the intersection of the tangent of the melting peak with the extrapolated baseline; ^(b)^ T_m1_: melting temperature; ^(c)^ ΔH_m1_: enthalpy of melting. Cooling cycle: ^(d^^)^ T_onset, cr_: the onset temperature defined as the intersection of the tangent of the crystallization peak with the extrapolated baseline; ^(e)^ T_cr_: crystallization temperature; ^(f)^ ΔH_cr_: enthalpy of crystallization. 2nd Heating cycle: ^(g)^ T_onset, m2_: the onset temperature defined as the intersection of the tangent of the melting peak with the extrapolated baseline; ^(h)^ T_m2_: melting temperature; ^(i)^ ΔH_m2_: enthalpy of melting; ^(l)^ T_g_: glass transition temperature; ^(m)^ n.d.: not detected.

**Table 7:** Preliminary evaluation of the effect of water contact on the macroscopic properties of H-PAA films (~20 °C).

Polymer	Apparent Flexibility	Resistance to Handling	Resistance after Contact with Water
B12-DM2	+	+	-
B12-DM6	+	+	+
B12-DE2	+	+	-
B12-PIP	-	-	-
B10-DM6	+	+	+
B8-DM6	+	+	+
B6-DM2	+	+	-
B6- DM6	+	+	+
B6-DE2	+	+	-
B6-PIP	-	-	+

**Table 8:** Tensile data of *b*B8-DM6 and *b*B12-DM6.

Sample	Thickness (mm)	Young Modulus (MPa)	Stress Max ^(a)^ (MPa)	Stress at Break (MPa)	Strain at Break (%)
*b*B8-DM6	0.10 ± 0.02	260 ± 24	3.9 ± 0.2	2.1 ± 0.5	1.7 ± 0.2
*b*B12-DM6	0.12 ± 0.02	267 ± 23	10.0 ± 0.3	5.9 ± 1.9	6.9 ± 0.3

^(a)^ Maximal value of the stress-strain curve.

**Table 9:** Wettability of H-PAAs.

Polymer Sample	Place	Contact Angle (deg)	Wetting Tension (dy/cm)	Base (mm)	Base Area (mm^2^)	Sessile Volume (μL)
*b*B8-DM6	Border	39.9	56.0	7.6	44.8	32.0
*b*B8-DM6	Center	40.3	55.7	7.3	40.9	28.4
*b*B12-DM6	Border	60.5	35.9	6.1	28.8	28.3
*b*B12-DM6	Center	54.2	42.7	6.7	33.9	31.0

## Data Availability

Not applicable.

## Supplementary Materials

The following are available online at https://www.mdpi.com/2073-4360/13/7/1018/s1, Figures S1–S3: ^1^H-NMR spectra of bis-acrylamides with assignments. Figures S4–S16: ^1^H-NMR spectra of H-PAAs with assignments. Figures S17–S29: FT-IR/ATR spectra of H-PAAs with assignments. Figure S30: TGA curves of B12-DM6 derived from solution and bulk synthesis in nitrogen (a) and air (b). Figures S31–S44: DSC thermograms of H-PAAs.
