# Supplementary material for: Semi-Crystalline Hydrophobic Polyamidoamines: A New Family of Technological Materials?
Source: Polymers (Basel). 2021 Mar 25;13(7):1018. doi: 10.3390/polym13071018 (PMC8036605; doi:10.3390/polym13071018)
Supplement: Supplementary file 1 [file polymers-13-01018-s001.pdf]

<u>Supplementary Materials</u>

Semi-Crystalline Hydrophobic Polyamidoamines: a New Family of Technological Materials?

Massimo Marcioni[1,2], Jenny Alongi[1], Elisabetta Ranucci[1], Mario Malinconico[3], Paola Laurienzo[3], Paolo Ferruti[1]* and Amedea Manfredi[1]*

[1] Dipartimento di Chimica, Università degli Studi di Milano, via C. Golgi 19, 20133 Milano, Italy; jenny.alongi@unimi.it (J.A.); elisabetta.ranucci@unimi.it (E.R.)

[2] Present address: Dipartimento di Scienza Applicata e Tecnologia, Politecnico di Torino, Alessandria campus, viale T. Michel, 15121 Alessandria, Italy; massimo.marcioni@polito.it (M.M.)

[3] Istituto Polimeri, Compositi e Biomateriali, Consiglio Nazionale delle Ricerche, via Campi Flegrei 34, 80078 Pozzuoli (NA), Italy; mario.malinconico@ipcb.cnr.it (M.M. CNR); paola.laurienzo@ipcb.cnr.it (P.L.)

* Correspondence: paolo.ferruti@unimi.it (P.F.); amedea.manfredi@unimi.it (A.M.); Tel.: +39-02-50314128 (P.F.); +39-02-50314181 (A.M.)

Pages S1-S47

Figures S1-S3: ^{1}H-NMR spectra of bisacrylamides with assignments.

Figures S4-S16: ^{1}H-NMR spectra of H-PAAs with assignments.

Figures S17-S29: FT-IR/ATR spectra of H-PAAs with assignments.

Figures S30: TG curves of B12-DM6 derived from solution and bulk synthesis in nitrogen (a) and air (b).

Figures S31-S44: DSC thermograms of H-PAAs.

^{1}H-NMR characterization

All PAAs were characterized by ^{1}H-NMR spectroscopy, using a Brüker Avance DPX-400 NMR spectrometer (Milano, Italy) operating at 400.13 MHz. Number of scans 32, relaxation delay, *d1*, 10.0 s, receiver gain automatically measured and set by the instrument. Analyses for bisacrylamides were performed in CD_3OD, for polymers in mixtures of deuterated solvents specified in figure captions.

B10

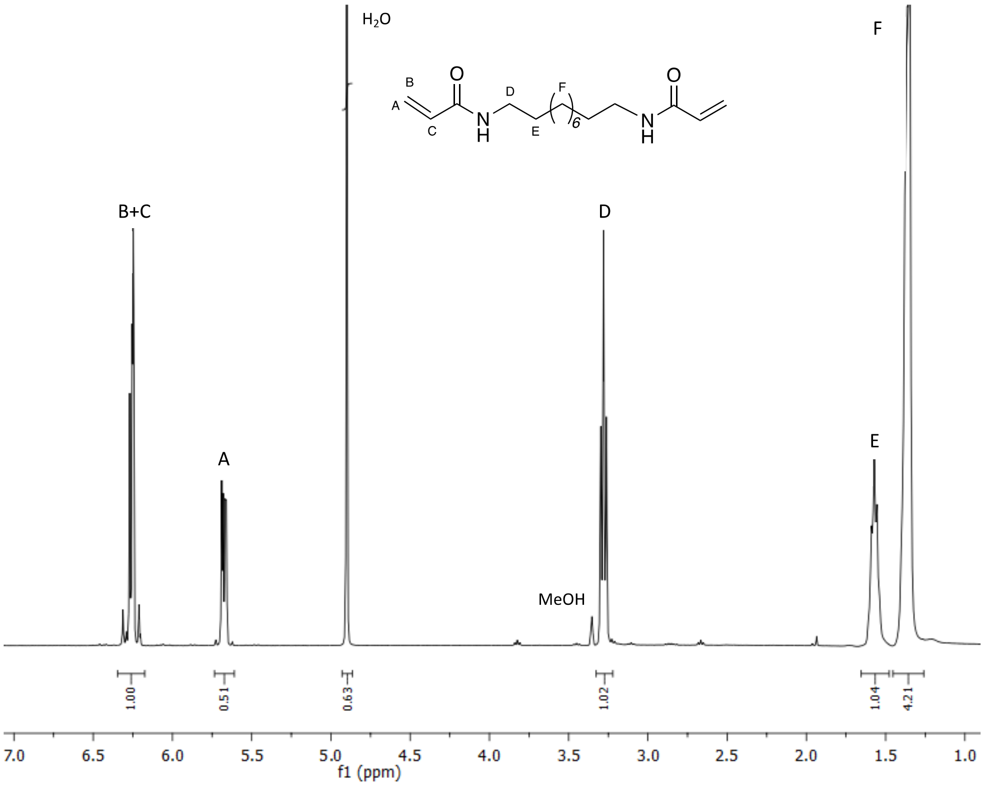

Figure S1. ^{1}H-NMR spectrum of B10.

^{1}H-NMR (CD_3OD) δ: 6.29-6.19 (m, 4H, B + C), 5.66-5.63 (m, 2H, A), 3.27-3.24 (t, 4H, D), 1.55-1.53 (bt, 4H, E), 1.32 (bs, 16H, F).

B8

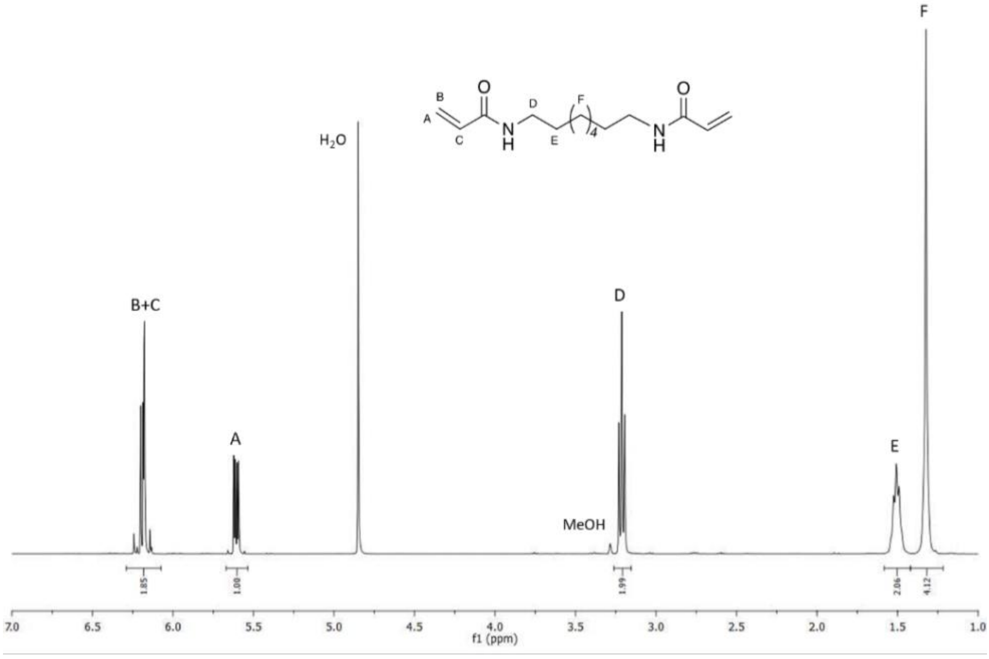

Figure S2. ^{1}H-NMR spectrum of B8.

^{1}H-NMR (CD_3OD) δ: 6.27-6.17 (m, 4H, B + C), 5.64-5.61 (m, 2H, A), 3.25-3.22 (t, 4H, D), 1.53 (bt, 4H, E), 1.35 (bs, 8H, F).

B6

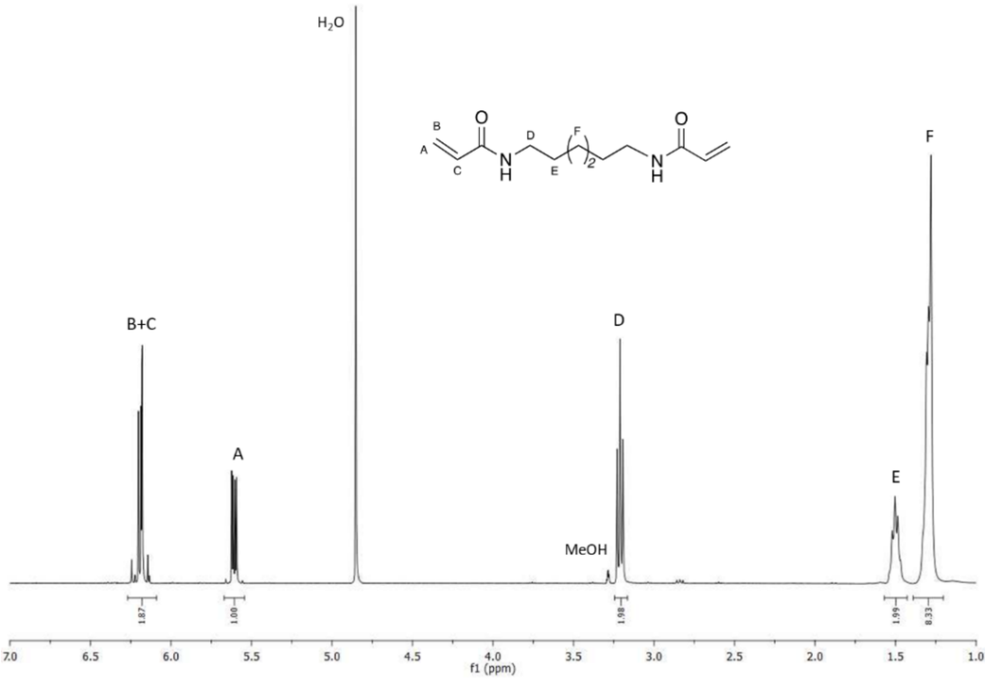

Figure S3. ^{1}H-NMR spectrum of B6.

^{1}H-NMR (CD_3OD) δ: 6.29-6.18 (m, 4H, B + C), 5.67-5.64 (m, 2H, A), 3.28-3.25 (t, 4H, D), 1.58-1.55 (t, 4H, D), 1.42-1.38 (m, 8H, F).

B12-DE2

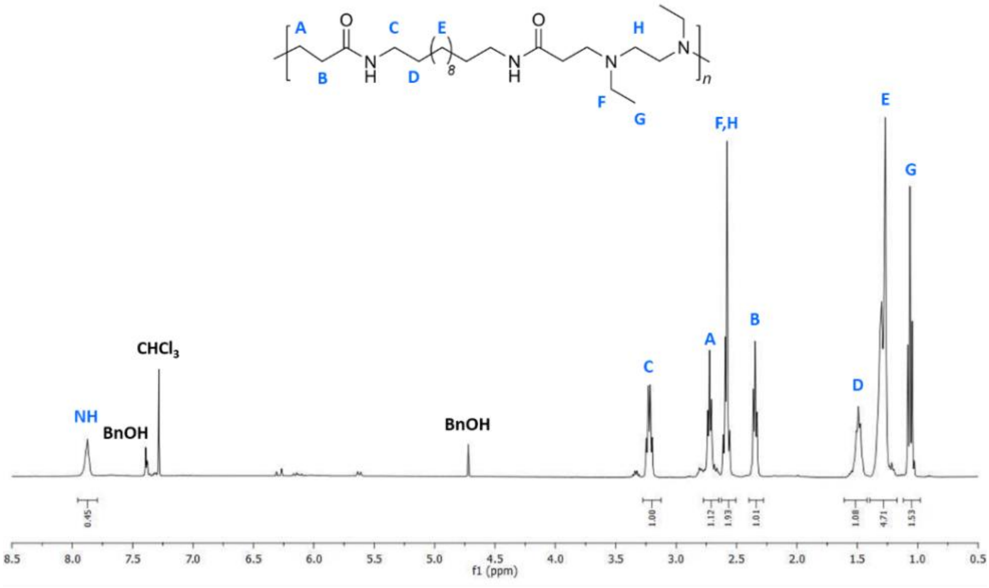

Figure S4. ^{1}H-NMR spectrum of B12-DE2.

^{1}H-NMR ($CDCl_3$) δ: 7.85 (s, 2H, NH), 3.22-3-17 (q, 4H, C), 2.72-2.69 (t, 4H, A), 2.57-2.53 (m, 8H, F,H), 2.34-2.31 (t, 4H, B), 1.48-1.45 (s, 4H, D), 1.28-1.25 (d, 16H, E), 1.06-1.02 (t, 6H, G).

B12-DB2

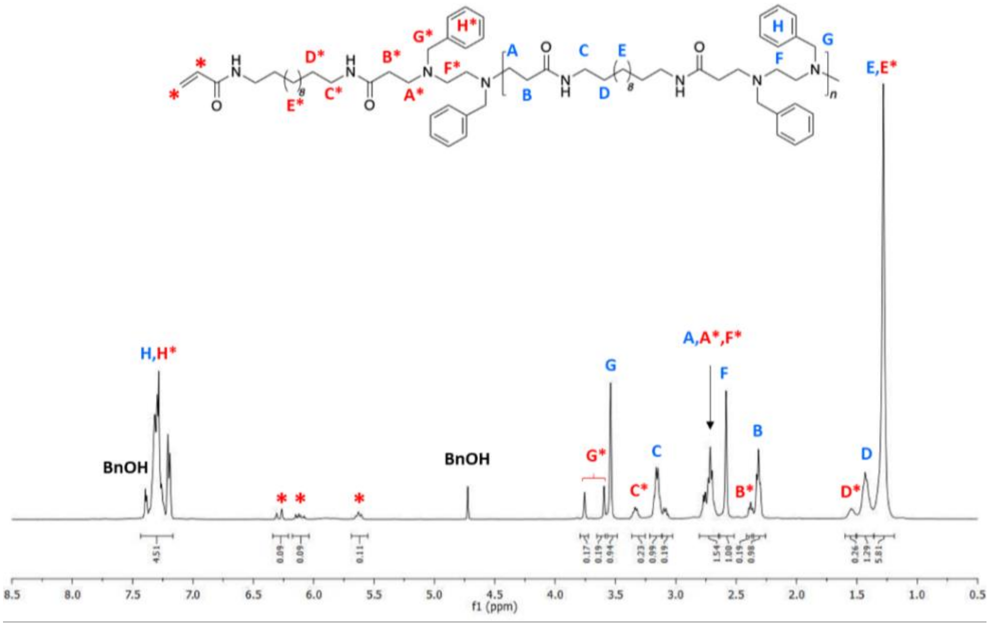

Figure S5. ^{1}H-NMR spectrum of B12-DB2.

^{1}H-NMR ($CDCl_3$) δ: 7.40-7.19 (m, 10H, H), 3.54 (s, 4H, G), 3.16-3.15 (d, 4H, C), 2.73-2.70 (t, 4H, A), 2.58 (s, 4H, F), 2.33-2.30 (t, 4H, B), 1.42 (bs, 4H, D), 1.28 (s, 16H, E).

B12-DM6

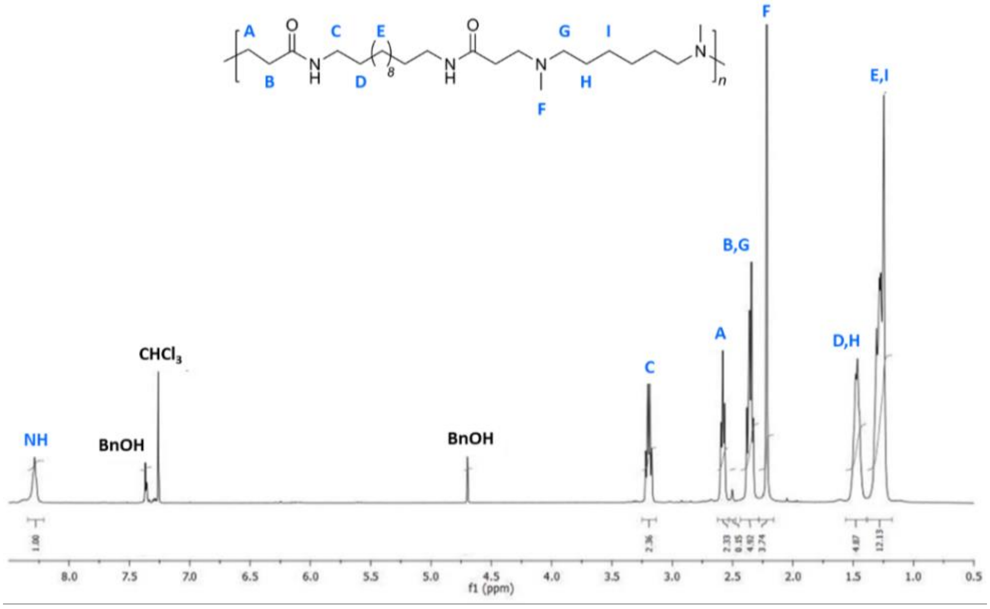

Figure S6. ^{1}H-NMR spectrum of B12-DM6.

^{1}H-NMR ($CDCl_3$) δ: 8.20 (s, 2H, NH), 3.20-3.17 (m, 4H, C), 2.60-2.57 (t, 4H, A), 2.38-2.33 (q, 8H, B,G), 2.22 (s, 6H, F), 1.48-1.45 (m, 8H, D,H), 1.31-1.25 (m, 20H, E,I).

B12-PIP

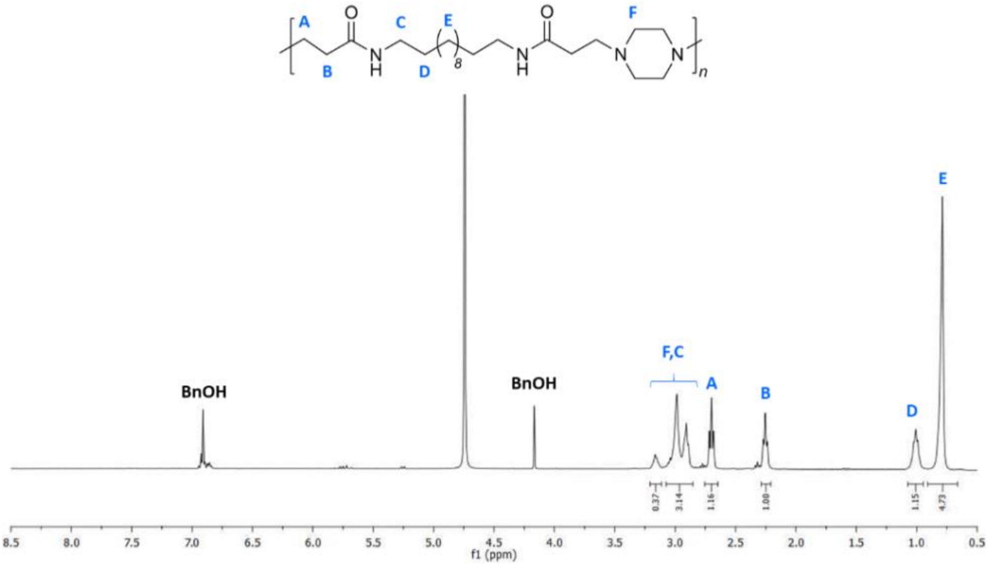

Figure S7. ^{1}H-NMR spectrum of B12-PIP.

^{1}H-NMR (D_2O/CD_3COOD) δ: 3.04-2.96 (m, 12H, C,F), 2.77-2.74 (t, 4H, A), 2.33-2.29 (t, 4H, B), 1.08-1.05 (bt, 4H, D), 0.84 (s, 16H, E).

B10-DM6

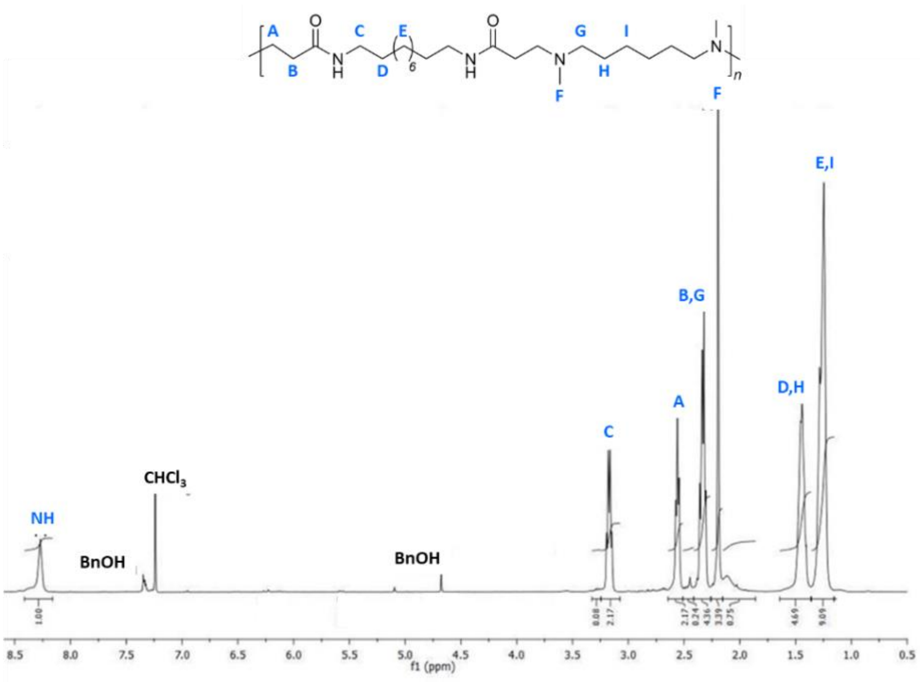

Figure S8. ^{1}H-NMR spectrum of B10-DM6.

^{1}H-NMR ($CDCl_3$) δ: 8.27 (bs, 2H, NH), 3.19-3.15 (q, 4H, C), 2.57-2.55 (t, 4H, A), 2.36-2.31 (m, 8H, B, G), 2.19 (s, 6H, F), 1.44 (m, 8H, D, H), 1.25 (m, 16H, E, I).

B8-DM6

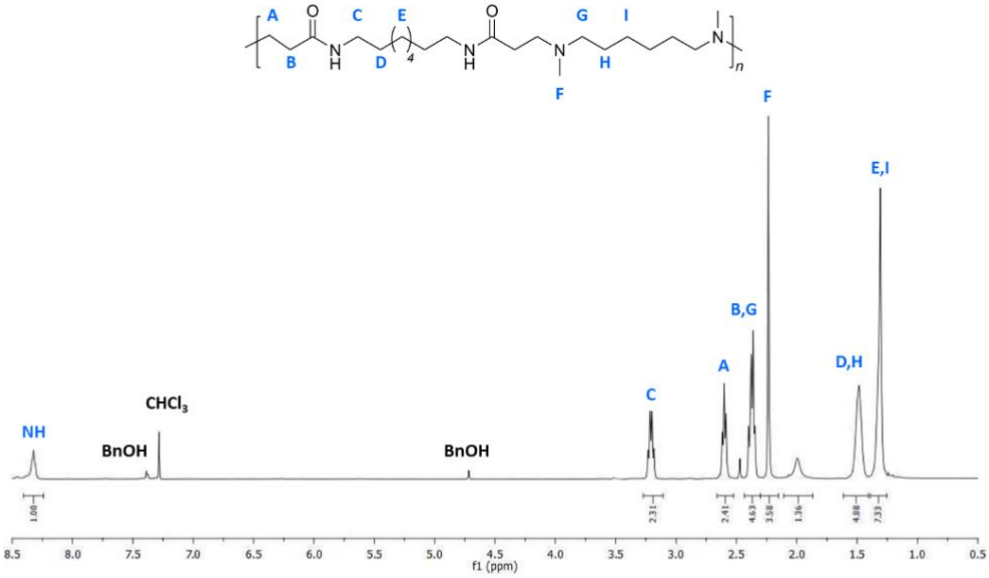

Figure S9. ^{1}H-NMR spectrum of B8-DM6.

^{1}H-NMR ($CDCl_3$) δ: 8.28 (bs, 2H, NH), 3.19-3.14 (q, 4H, C), 2.57-2.54 (t, 4H, A), 2.35-2.30 (m, 8H, B, G), 2.19 (s, 6H, F), 1.44 (bs, 8H, D, H), 1.26 (bs, 12H, E, I).

B6-DM2

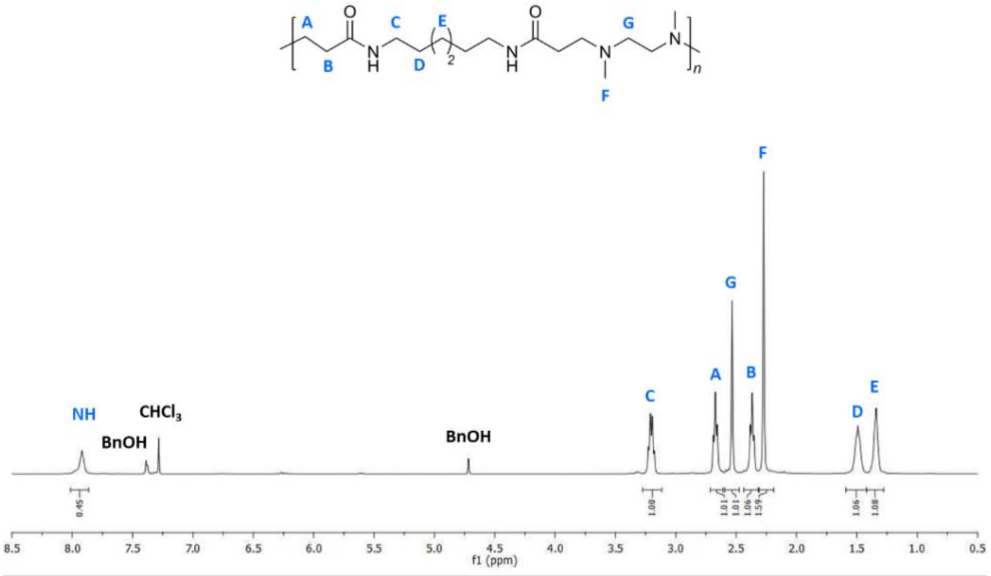

Figure S10. ^{1}H-NMR spectrum of B6-DM2.

^{1}H-NMR ($CDCl_3$) δ: 7.92 (bs, 2H, NH), 3.20-3.17 (q, 4H, C), 2.76-2.61 (t, 4H, A), 2.53 (s, 4H, G), 2.43-2.32 (t, 4H, B), 2.27 (s, 6H, F), 1.57-1.43 (bs, 4H, D), 1.41-1.24 (bs, 4H, E).

B6-DE2

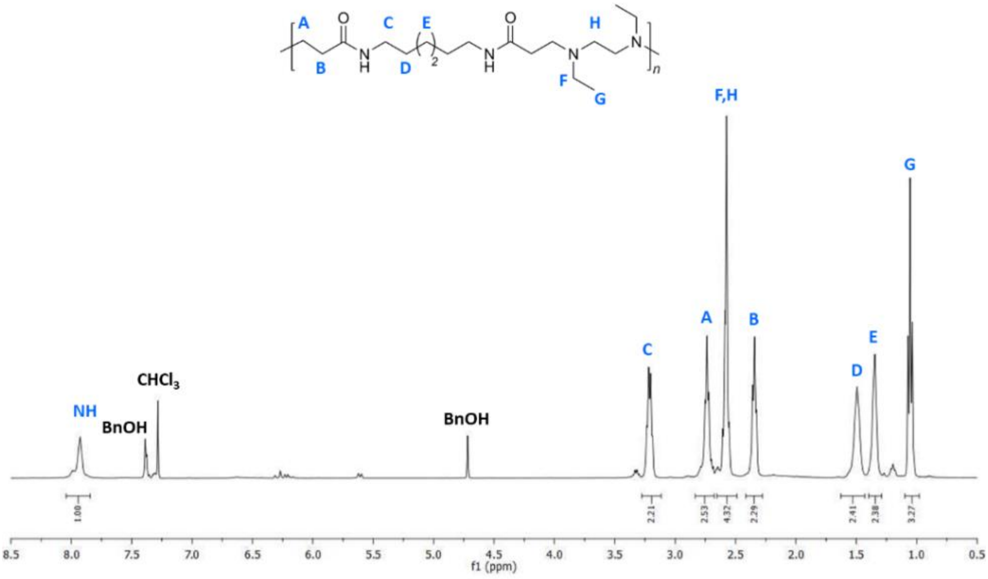

Figure S11. ^{1}H-NMR spectrum of B6-DE2.

^{1}H-NMR ($CDCl_3$) δ: 7.88 (bs, 2H, NH), 3.18-3.16 (q, 4H, C), 2.71-2.68 (t, 4H, A), 2.56-2.51 (m, 8H, F, H), 2.31-2.28 (t, 4H, B), 1.45 (bs, 4H, D), 1.30 (bs, 4H, E), 1.03-0.99 (t, 6H, G).

B6-DB2

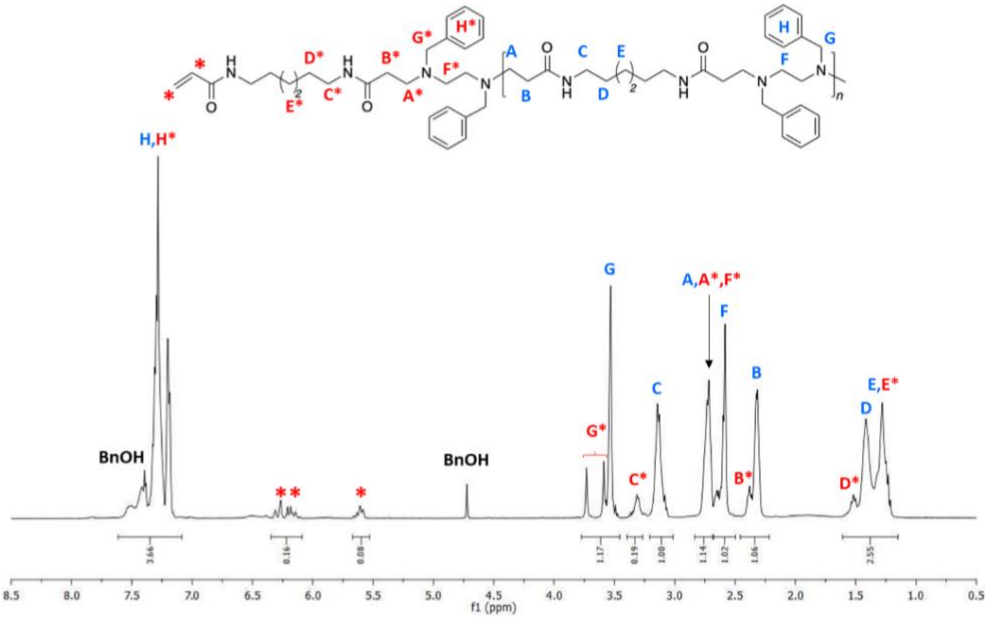

Figure S12. ^{1}H-NMR spectrum of B6-DB2.

^{1}H-NMR ($CDCl_3$) δ: 7.51-7.19 (m, 10H, H), 3.53 (s, 4H, G), 3.14-3.13 (m, 4H, C), 2.73-2.72 (m, 4H, A), 2.58 (m, 4H, F), 2.32-2.31 (m, 4H, B), 1.41-1.23 (m, 8H, D, E).

B6-DM6

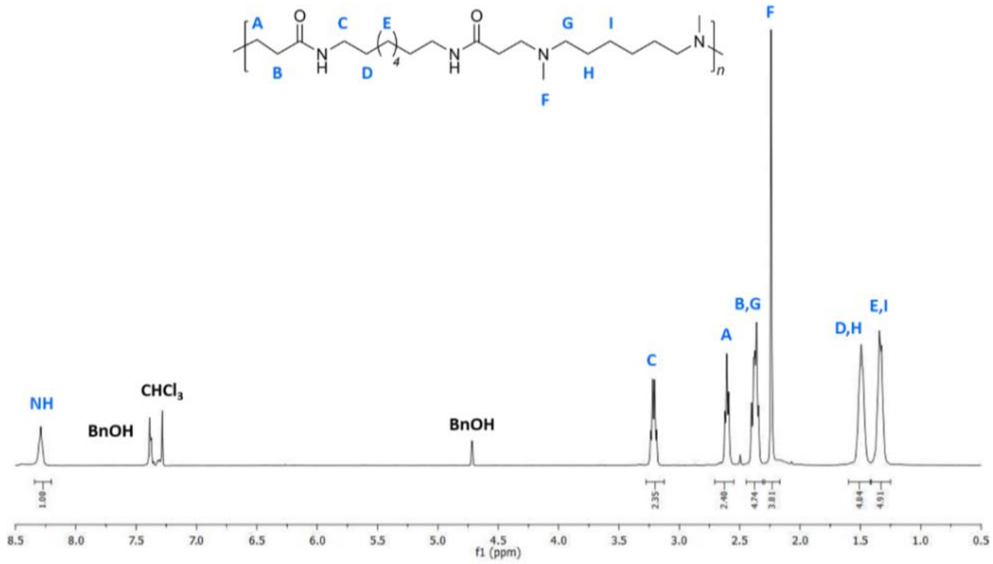

Figure S13. ^{1}H-NMR spectrum of B6-DM6.

^{1}H-NMR ($CDCl_3$) δ: 8.25 (bs, 2H, NH), 3.19-3.15 (q, 4H, C), 2.58-2.55 (t, 4H, A), 2.36-2.30 (m, 8H, B, G), 2.20 (s, 6H, F), 1.45 (s, 8H, D, H), 1.30 (m, 8H, E, I).

B6-PIP

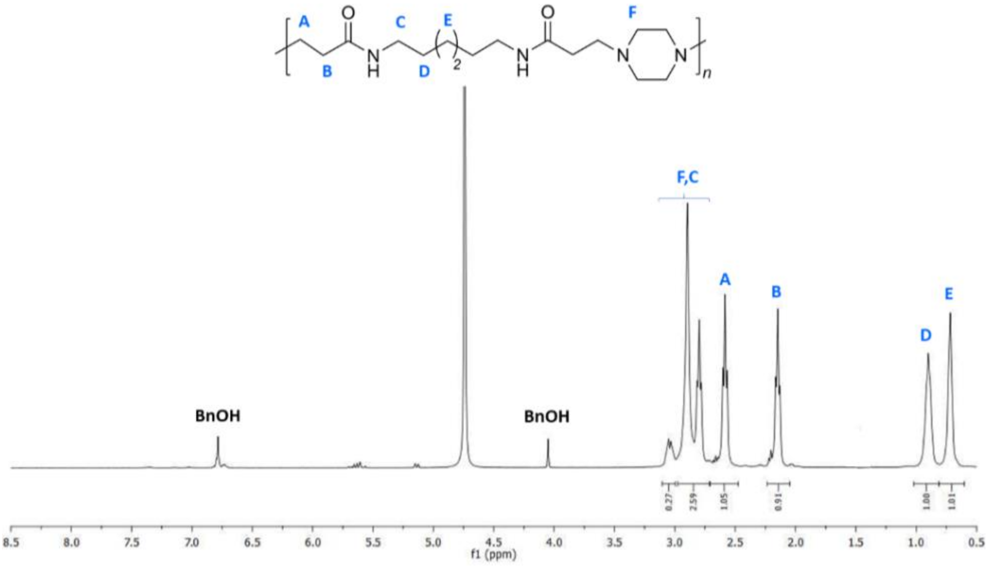

Figure S14. ^{1}H-NMR of B6-PIP.

^{1}H-NMR (D_2O/CD_3COOD) δ: 3.11 (m, 12H, F, C), 2.51-2.48 (t, 4H, A), 2.07-2.04 (t, 4H, B), 0.81 (bs, 4H, D), 0.63 (bs, 4H, E).

*b*B12-DM6

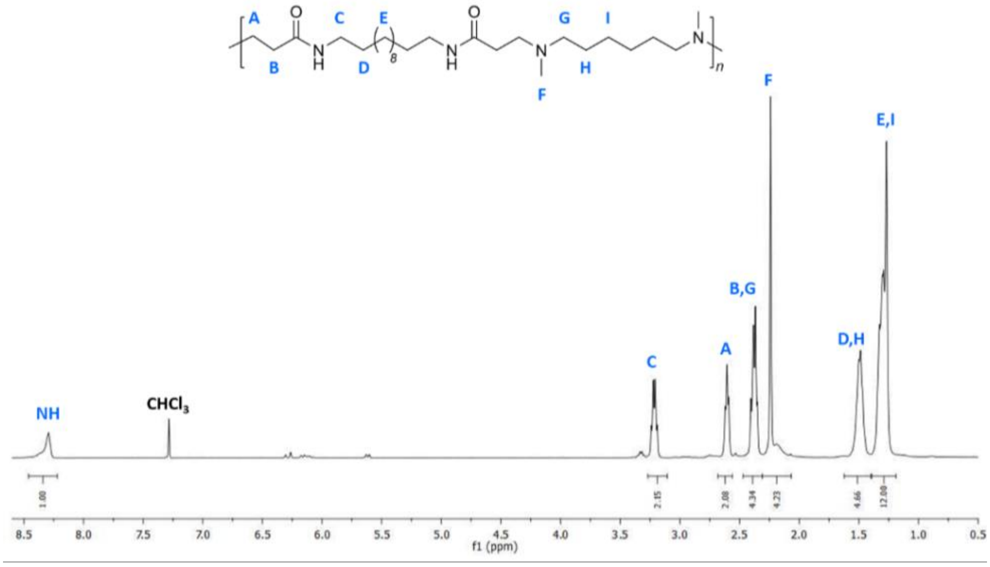

Figure S15. ^{1}H-NMR spectrum of B12-DM6 from bulk synthesis.

^{1}H-NMR ($CDCl_3$) δ: 8.30 (bs, 2H, NH), 3.22-3.21 (q, 4H, C), 2.60 (t, 4H, A), 2.36-2.38 (m, 8H, B, G), 2.23 (s, 6H, F), 1.49-1.35 (m, 8H, D, H), 1.30-1.28 (m, 20H, E, I).

*b*B8-DM6

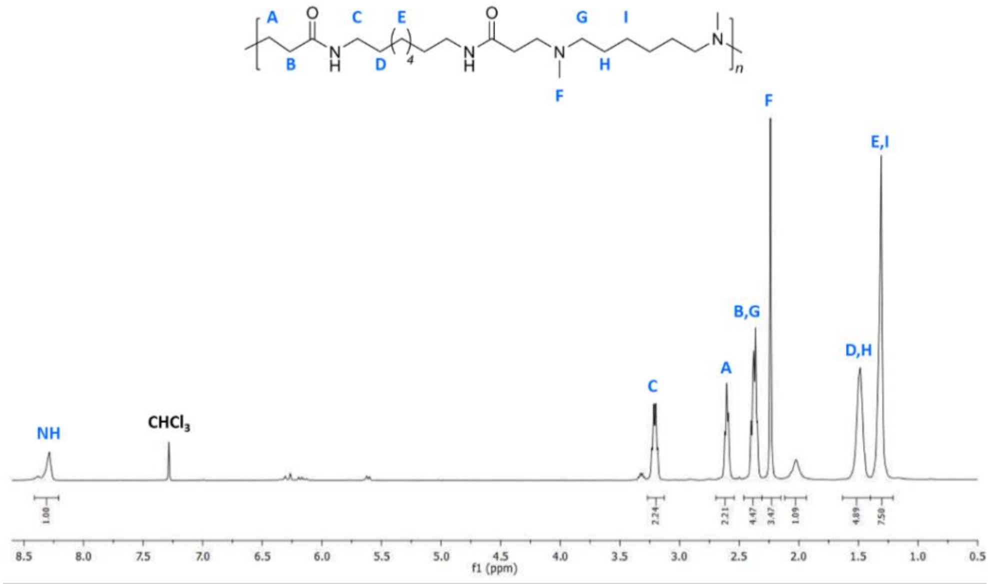

Figure S16. ^{1}H-NMR spectrum of B8-DM6 from bulk synthesis.

^{1}H-NMR ($CDCl_3$) δ: 8.29 (bs, 2H, NH), 3.25-3.16 (q, 4H, C), 2.68-2.55 (t, 4H, A), 2.45-2.30 (m, 8H, B, G), 2.24 (s, 6H, F), 1.61-1.41 (bs, 8H, D, H), 1.40-1.21 (bs, 12H, E, I).

FT-IR/ATR characterization

All PAAs were analyzed by attenuated total reflectance (ATR) Fourier transform infrared spectroscopy (FT-IR). FT-IR/ATR spectra were recorded at room temperature, in the 4000 - 380 cm^{-1} wavenumber range, with 32 scans and 4 cm^{-1} resolution using a Perkin-Elmer Frontier FT-IR/FIR spectrophotometer (Milano, Italy), equipped with a diamond crystal characterized by a penetration depth of 1.66 μm.

B12-DM2

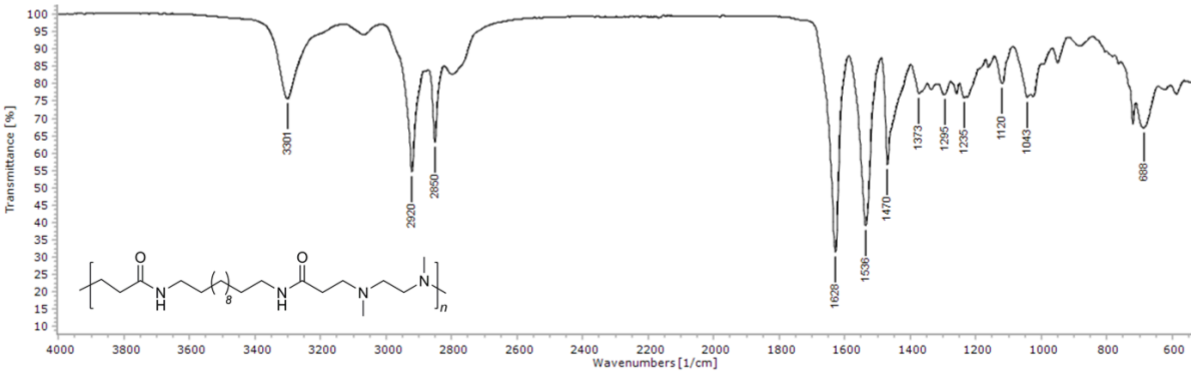

Figure S17. FT-IR/ATR spectrum of B12-DM2.

Assignments: 3301 cm^{-1} (N-H stretching), 2920 and 2850 cm^{-1} (C-H stretching), 1628 cm^{-1} (C=O stretching), 1536 cm^{-1} (N-H bending), 1470, 1373 and 1295 cm^{-1} (CH_2 bending), 1235 and 1043 cm^{-1} (C-N stretching), 1120 and 688 cm^{-1} (C-C bending).

B12-DE2

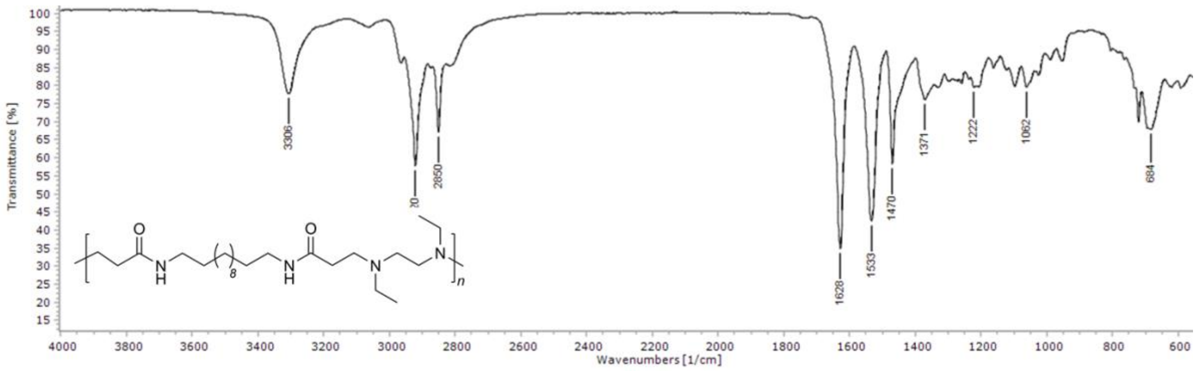

Figure S18. FT-IR/ATR spectrum of B12-DE2.

Assignments: 3306 cm^{-1} (N-H stretching), 2920 and 2850 cm^{-1} (C-H stretching), 1628 cm^{-1} (C=O stretching), 1533 cm^{-1} (N-H bending), 1470 and 1371 cm^{-1} (CH_2 bending), 1222 and 1062 cm^{-1} (C-N stretching), 684 cm^{-1} (C-C bending).

B12-DB2

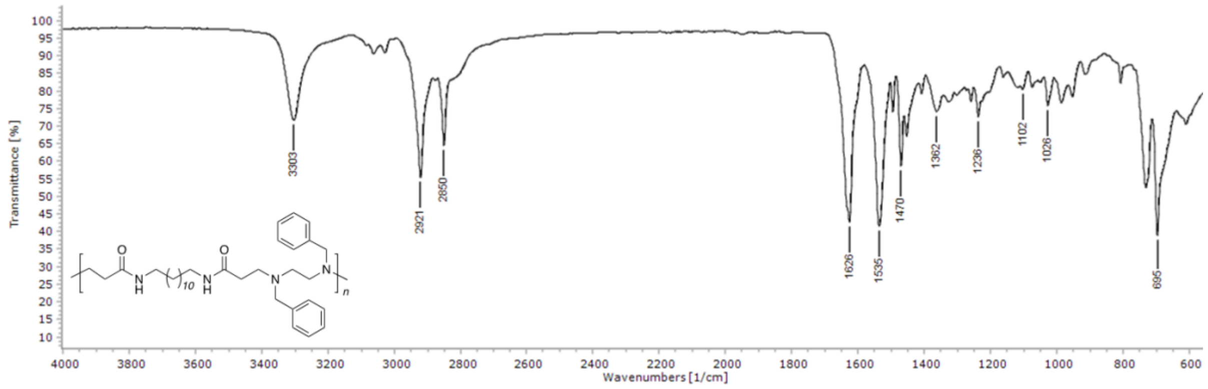

Figure S19. FT-IR/ATR spectrum of B12-DB2.

Assignments: 3303 cm^{-1} (N-H stretching), 2921 and 2850 cm^{-1} (C-H stretching), 1636 cm^{-1} (C=O stretching), 1535 cm^{-1} (N-H bending), 1470 and 1382 cm^{-1} (CH_2 bending), 1236 and 1026 cm^{-1} (C-N stretching), 1102 and 695 cm^{-1} (C-C bending).

B12-DM6

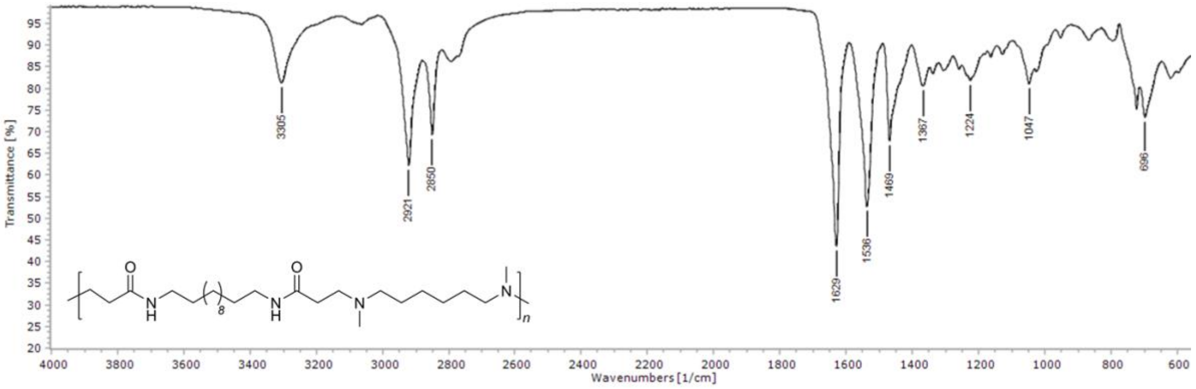

Figure S20. FT-IR/ATR spectrum of B12-DM6.

Assignments: 3305 cm^{-1} (N-H stretching), 2921 and 2850 cm^{-1} (C-H stretching), 1629 cm^{-1} (C=O stretching), 1536 cm^{-1} (N-H bending), 1469 and 1367 cm^{-1} (CH_2 bending), 1224 and 1047 cm^{-1} (C-N stretching), 696 cm^{-1} (C-C bending).

B12-PIP

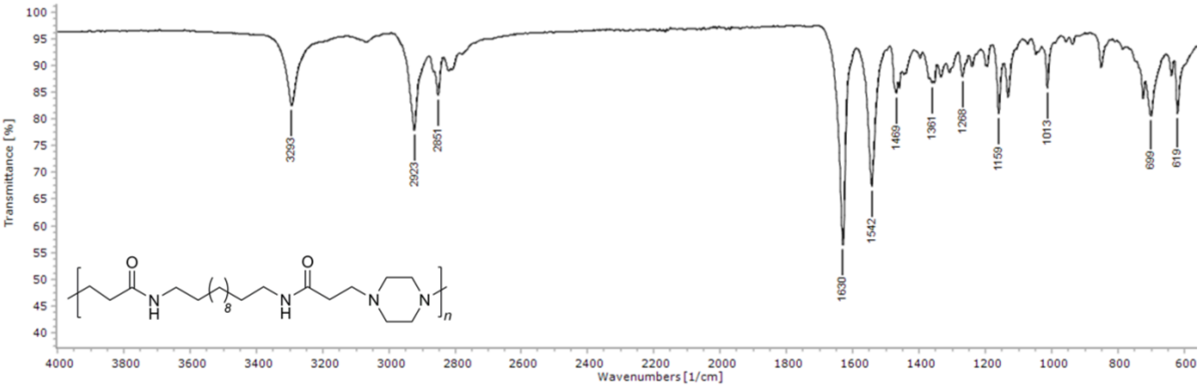

Figure S21. FT-IR/ATR spectrum of B12-PIP.

Assignments: 3293 cm^{-1} (N-H stretching), 2923, 2951 and 2810 cm^{-1} (C-H stretching), 1630 cm^{-1} (C=O stretching), 1542 cm^{-1} (N-H bending), 1469 and 1361 cm^{-1} (CH_2 bending), 1268 and 1013 cm^{-1} (C-N stretching), 1159, 699 and 619 cm^{-1} (C-C bending).

B10-DM6

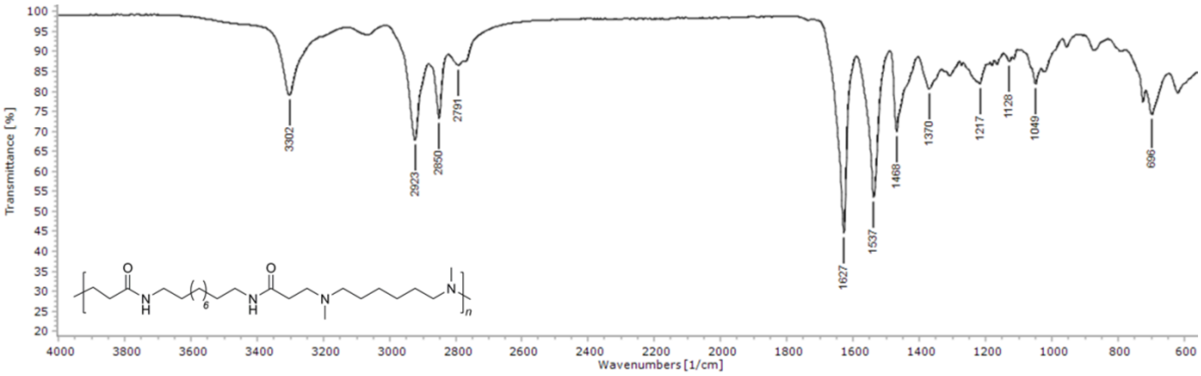

Figure S22. FT-IR/ATR spectrum of B10-DM6.

Assignments: 3302 cm^{-1} (N-H stretching), 2923, 2850 and 2791 cm^{-1} (C-H stretching), 1627 cm^{-1} (C=O stretching), 1537 cm^{-1} (N-H bending), 1468 and 1370 cm^{-1} (CH_2 bending), 1224 and 1049 cm^{-1} (C-N stretching), 696 cm^{-1} (C-C bending).

B8-DM6

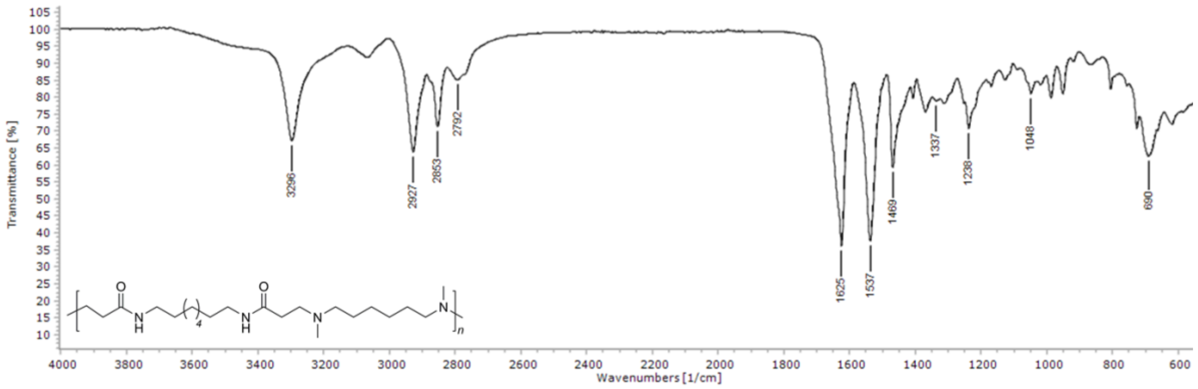

Figure S23. FT-IR/ATR spectrum of B8-DM6.

Assignments: 3300 cm^{-1} (N-H stretching), 2927, 2851 and 2789 cm^{-1} (C-H stretching), 1636 cm^{-1} (C=O stretching), 1536 cm^{-1} (N-H bending), 1467 and 1370 cm^{-1} (CH_2 bending), 1217 and 1048 cm^{-1} (C-N stretching), 698 cm^{-1} (C-C bending).

B6-DM2

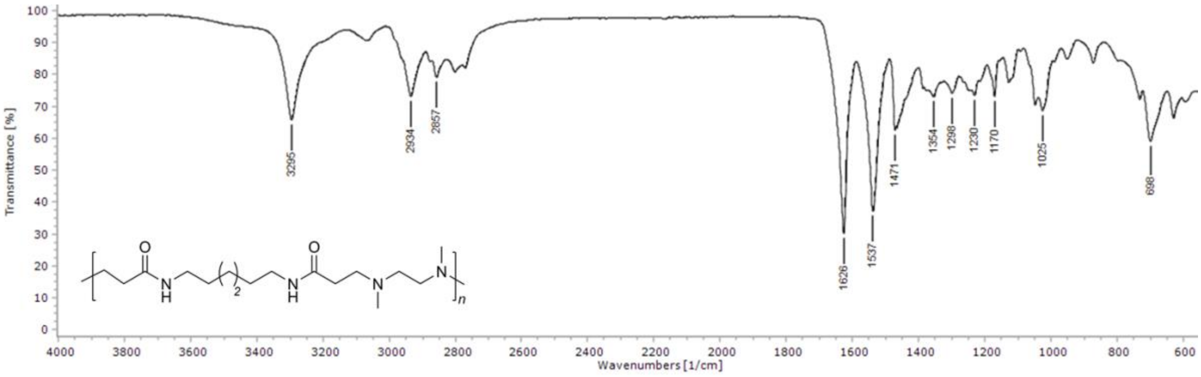

Figure S24. FT-IR/ATR spectrum of B6-DM2.

Assignments: 3297 cm^{-1} (N-H stretching), 2932 and 2855 cm^{-1} (C-H stretching), 1626 cm^{-1} (C=O stretching), 1537 cm^{-1} (N-H bending), 1461, 1365 and 1306 cm^{-1} (CH_2 bending), 1227 and 1043 cm^{-1} (C-N stretching), 1178 and 698 cm^{-1} (C-C bending).

B6-DE2

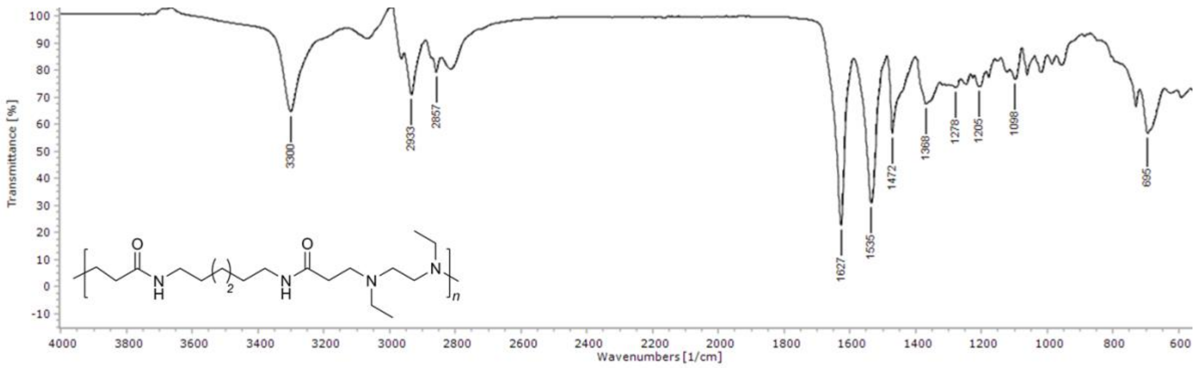

Figure S25. FT-IR/ATR spectrum of B6-DE2.

Assignments: 3300 cm^{-1} (N-H stretching), 2933 and 2857 cm^{-1} (C-H stretching), 1627 cm^{-1} (C=O stretching), 1535 cm^{-1} (N-H bending), 1472 and 1368 cm^{-1} (CH_2 bending), 1205 cm^{-1} (C-N stretching), 1098 and 695 cm^{-1} (C-C bending).

B6-DM6

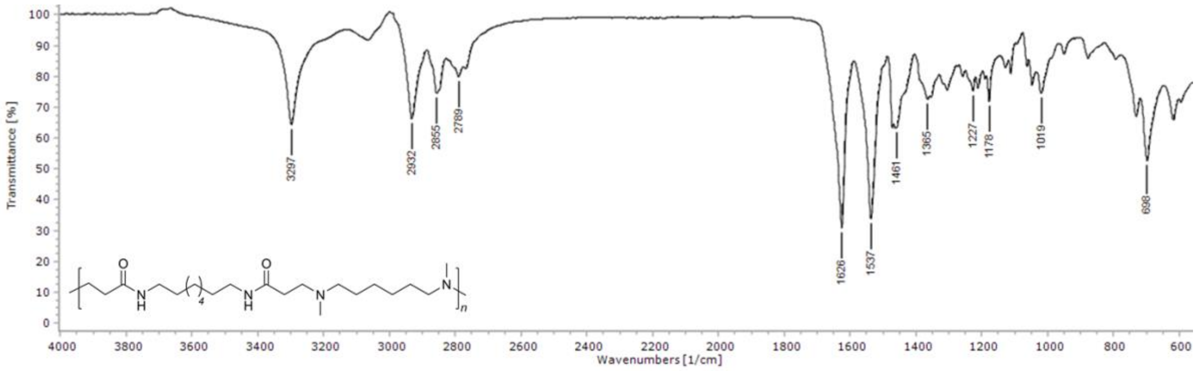

Figure S26. FT-IR/ATR spectrum of B6-DM6.

Assignments: 3297 cm^{-1} (N-H stretching), 2932, 2855 and 2789 cm^{-1} (C-H stretching), 1626 cm^{-1} (C=O stretching), 1537 cm^{-1} (N-H bending), 1461 and 1365 cm^{-1} (CH_2 bending), 1227 and 1019 cm^{-1} (C-N stretching), 698 cm^{-1} (C-C bending).

B6-PIP

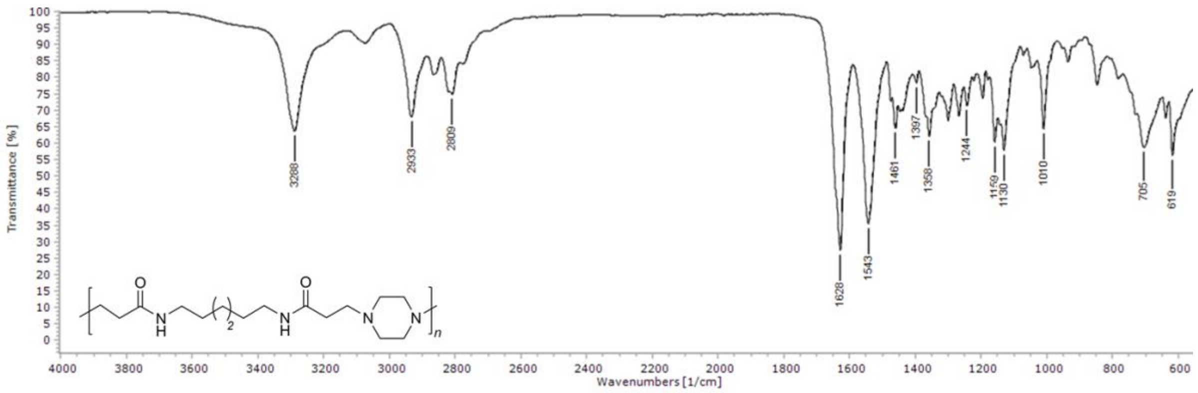

Figure S27. FT-IR/ATR of B6-PIP.

Assignments: 3288 cm^{-1} (N-H stretching), 2933, 2951 and 2809 cm^{-1} (C-H stretching), 1628 cm^{-1} (C=O stretching), 1543 cm^{-1} (N-H bending), 1461 and 1358 cm^{-1} (CH_2 bending), 1244 and 1010 cm^{-1} (C-N stretching), 1130, 705 and 619 cm^{-1} (C-C bending).

*b*B12-DM6

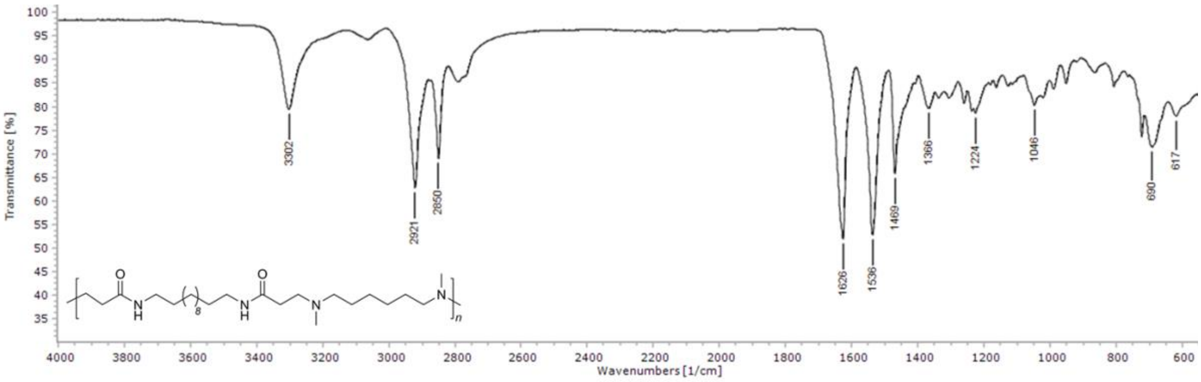

Figure S28. FT-IR/ATR spectrum of B12-DM6 from bulk synthesis.

Assignments: 3302 cm^{-1} (N-H stretching), 2921 and 2850 cm^{-1} (C-H stretching), 1626 cm^{-1} (C=O stretching), 1536 cm^{-1} (N-H bending), 1469 and 1366 cm^{-1} (CH_2 bending), 1224 and 1046 cm^{-1} (C-N stretching), 690 and 618 cm^{-1} (C-C bending).

*b*B8-DM6

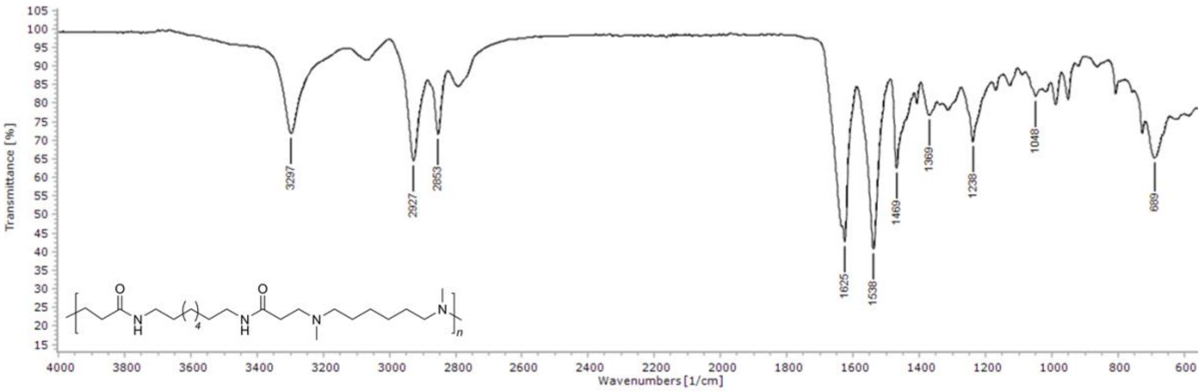

Figure S29. FT-IR/ATR spectrum of B8-DM6 from bulk synthesis.

Assignments: 3297 cm^{-1} (N-H stretching), 2927 and 2853 cm^{-1} (C-H stretching), 1625 cm^{-1} (C=O stretching), 1538 cm^{-1} (N-H bending), 1469 and 1369 cm^{-1} (CH_2 bending), 1238 and 1048 cm^{-1} (C-N stretching), 689 cm^{-1} (C-C bending).

TGA characterization

TGA analyses were performed in nitrogen and air, respectively, from 30 to 800 °C with a heating rate of 10 °C min^{-1}. A Mettler-Toledo (Milano, Italy) thermogravimetric balance, TGA/DSC 2 Star® System, was used, placing samples (5 mg) in open alumina pans, in an inert or oxidative atmosphere (50 mL min^{-1} gas flow).

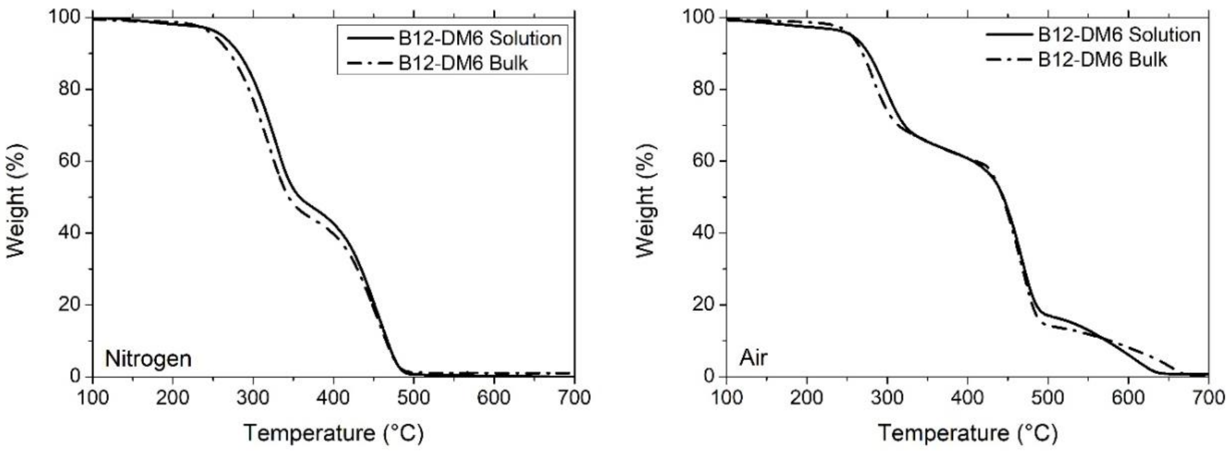

Figure S30. TG curves of B12-DM6 derived from solution and bulk synthesis in nitrogen (a) and air (b).

DSC characterization

DSC analysis were performed on a Mettler-Toledo DSC823^e System. In detail, 5 mg sample was placed in a standard Al pan and heated or cooled at 5 °C min^{-1} under 80 mL min^{-1} nitrogen flow, following the step of heating/cooling cycles:

- 1st step: heating from 25 °C to T_f, where T_f is the final T that is different for all polymers
- 2nd step: 1 min at T_f
- 3rd step: cooling from T_f to 25 °C
- 4th step: 1 min at 25 °C
- 5th step: heating from 25 °C to T_f

T_g values were assessed by a TA Waters instrument Model DSCQ20 (Milano, Italy): samples (5.5 mg) were placed in standard aluminum crucibles and heated or cooled at 10 °C min^{-1} under 80 mL min^{-1} nitrogen flow. The heating program was as follows: 1st step: heating from 25 °C to T_f; 2nd step: 1 min at T_f; 3rd step: cooling from T_x to -50 °C; 4th step: 1 min at -50 °C; 5th step: heating from -50 °C to T_x.

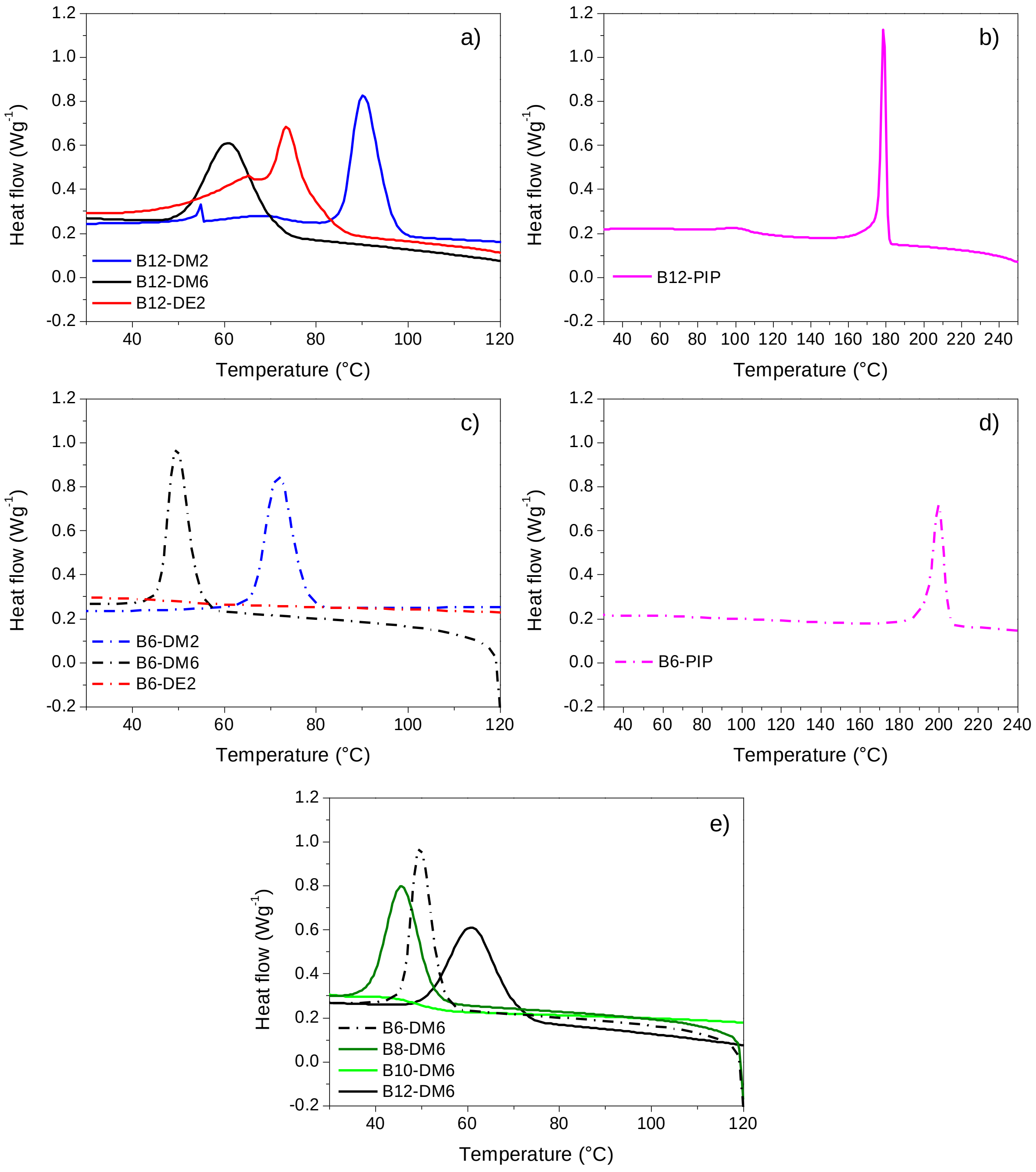

Figure S31. DSC thermograms of H-PAAs pertaining to cooling cycle at 10 °C min^{-1}.

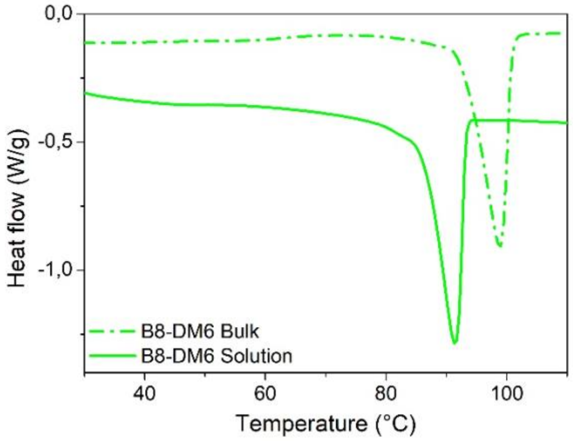

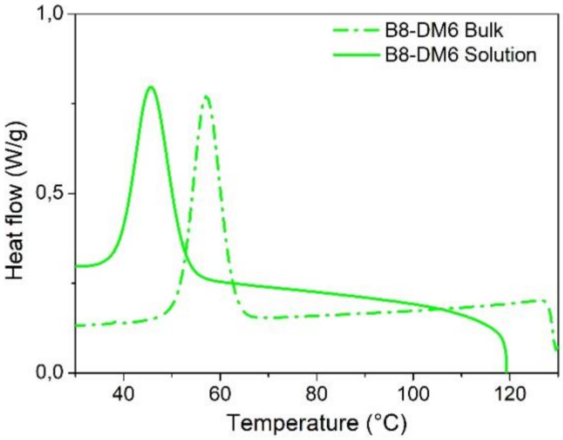

Figure S32. DSC thermograms of B8-DM6 samples obtained by synthesis in solution and bulk; panel a) 2nd heating cycle and panel b) cooling cycle.

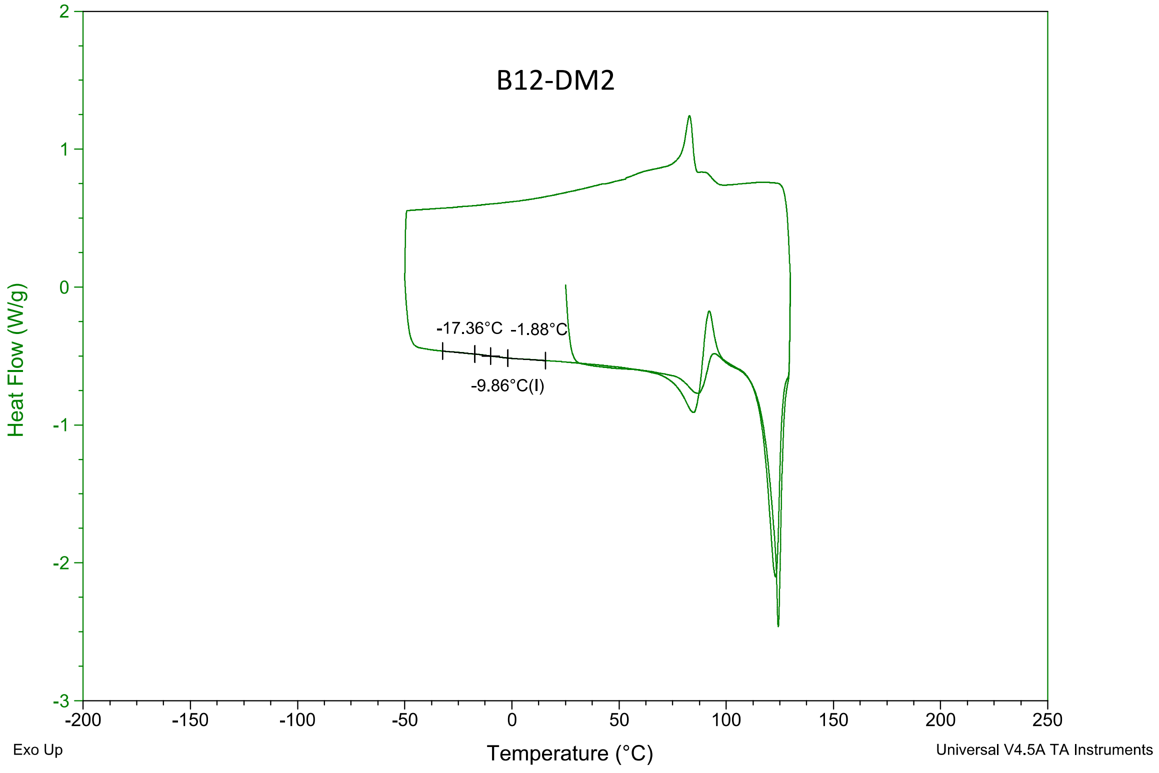

Figure S33. DSC thermograms of B12-DM2 for T_g evaluation.

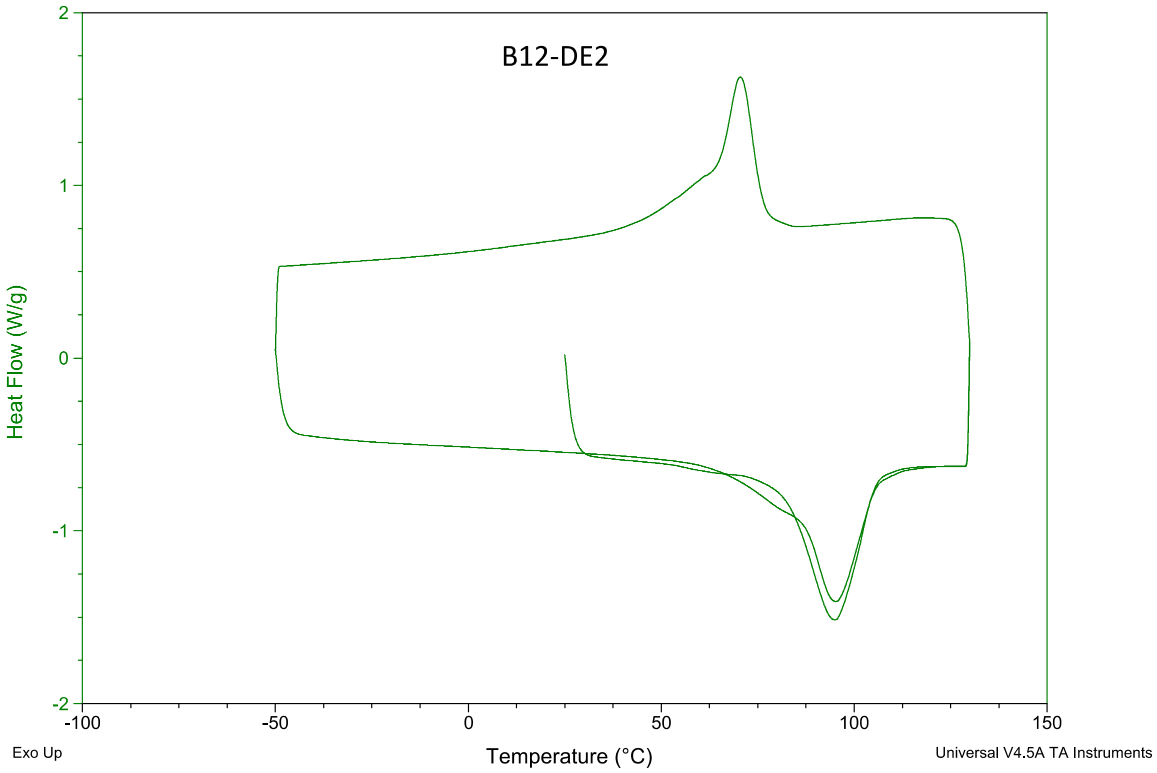

Figure S34. DSC thermograms of B12-DE2 for T_g evaluation.

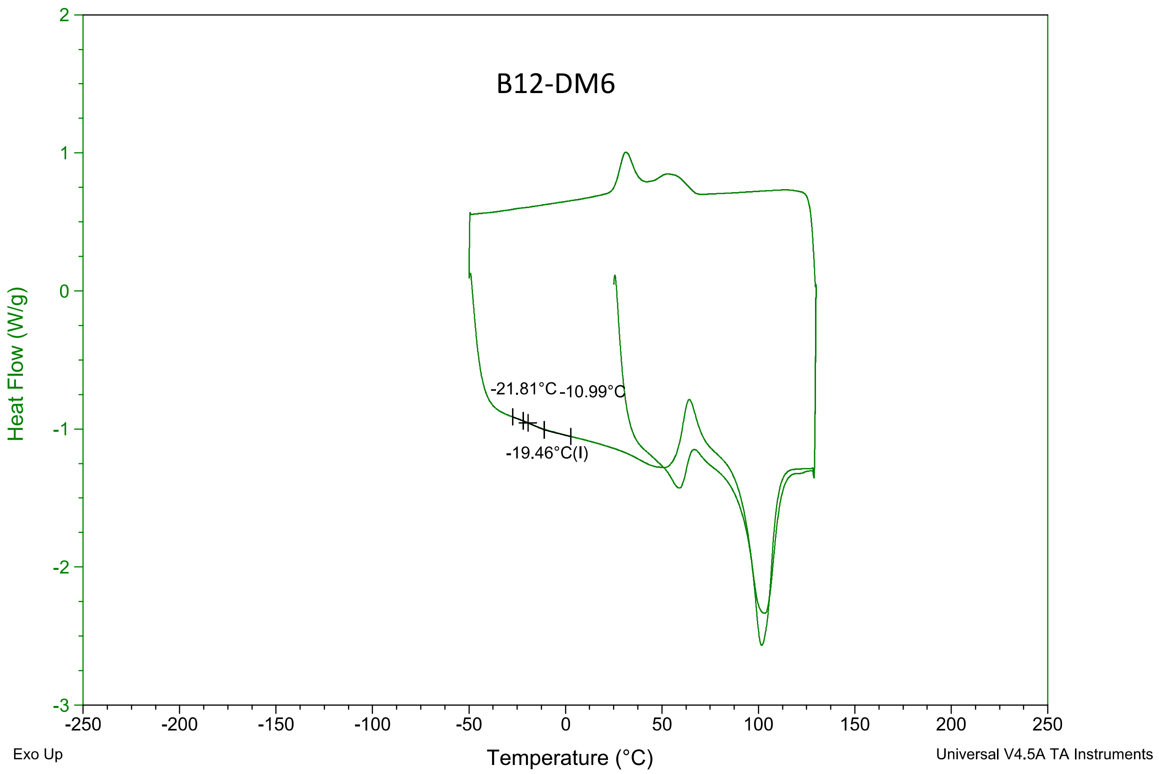

Figure S35. DSC thermograms of B12-DM6 for T_g evaluation.

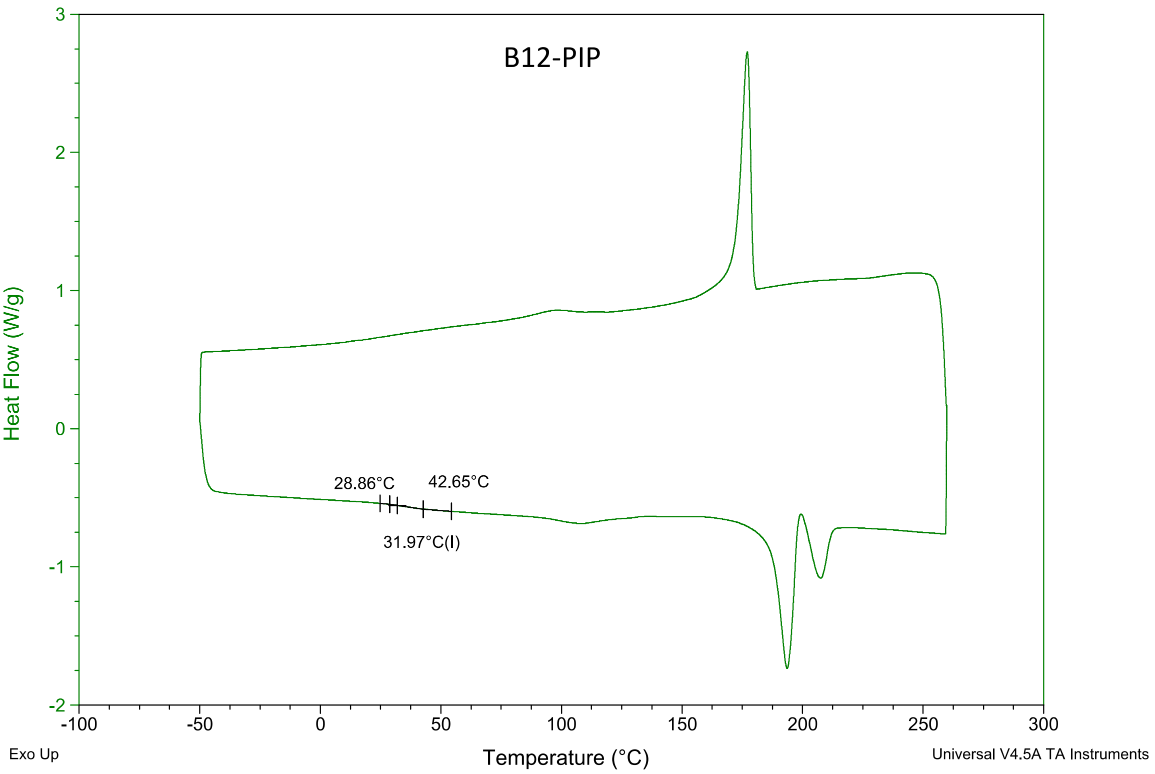

Figure S36. DSC thermograms of B12-PIP for T_g evaluation.

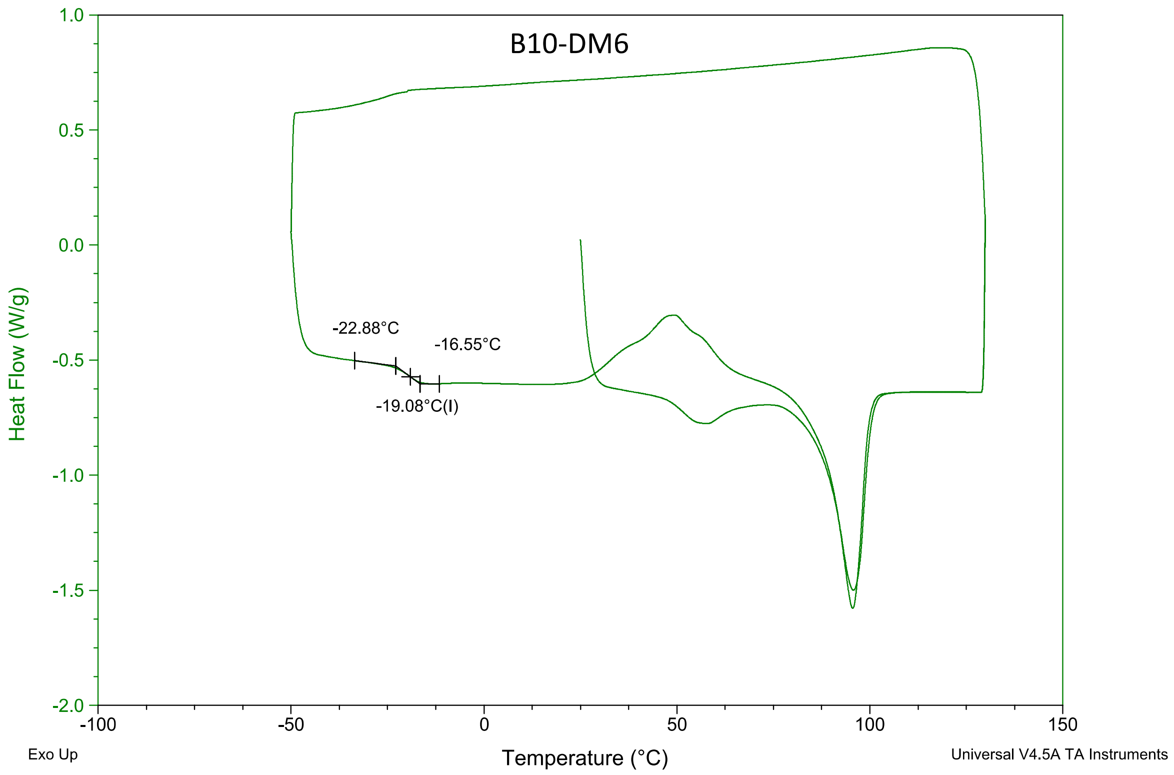

Figure S37. DSC thermograms of B10-DM6 for T_g evaluation.

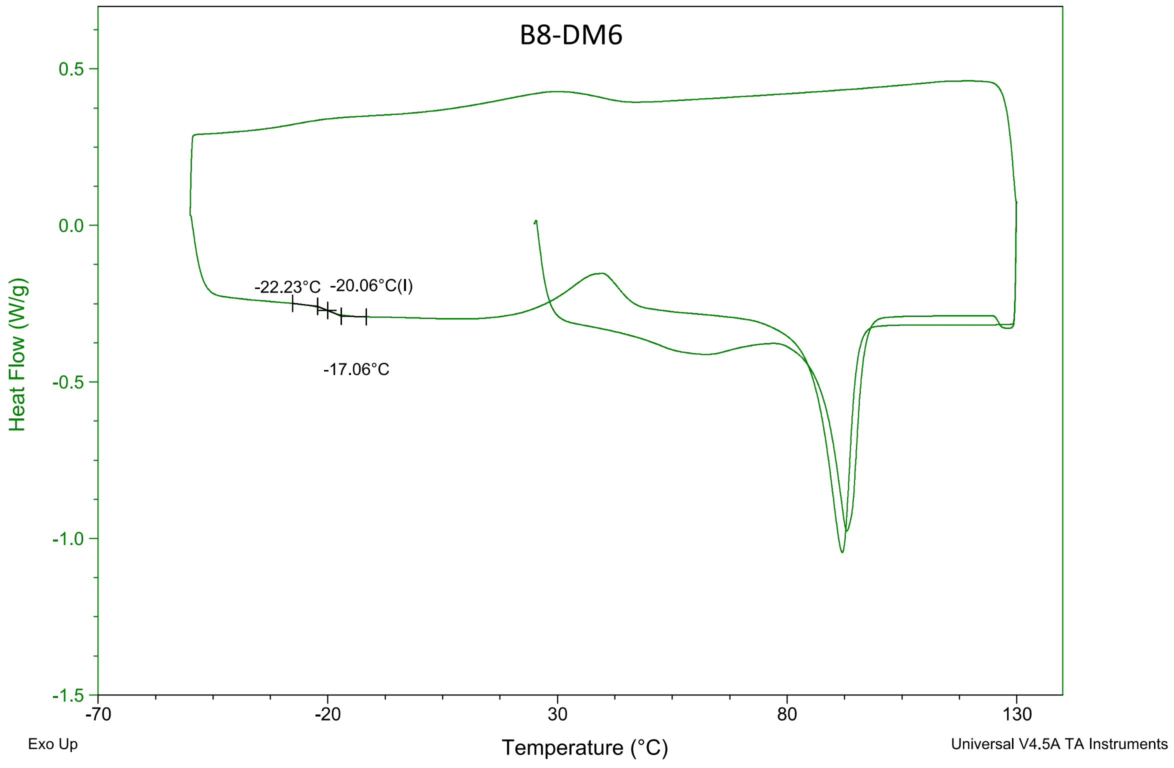

Figure S38. DSC thermograms of B8-DM6 for T_g evaluation.

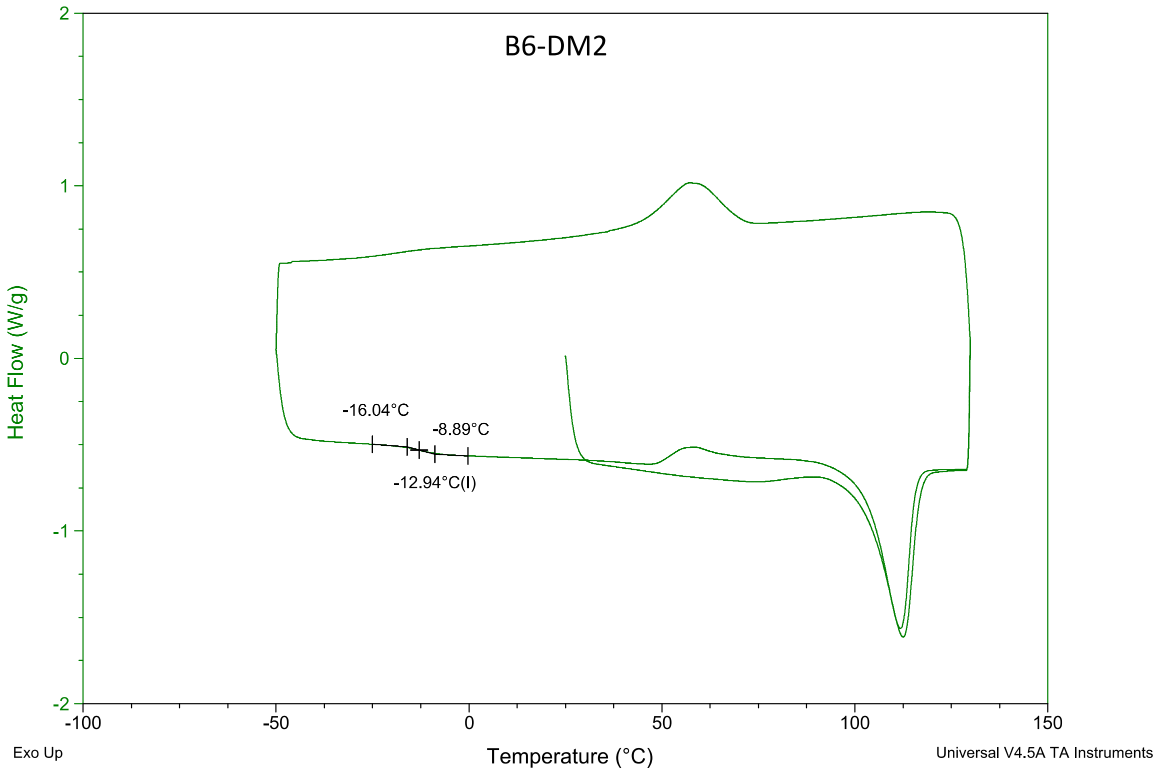

Figure S39. DSC thermograms of B6-DM2 for T_g evaluation.

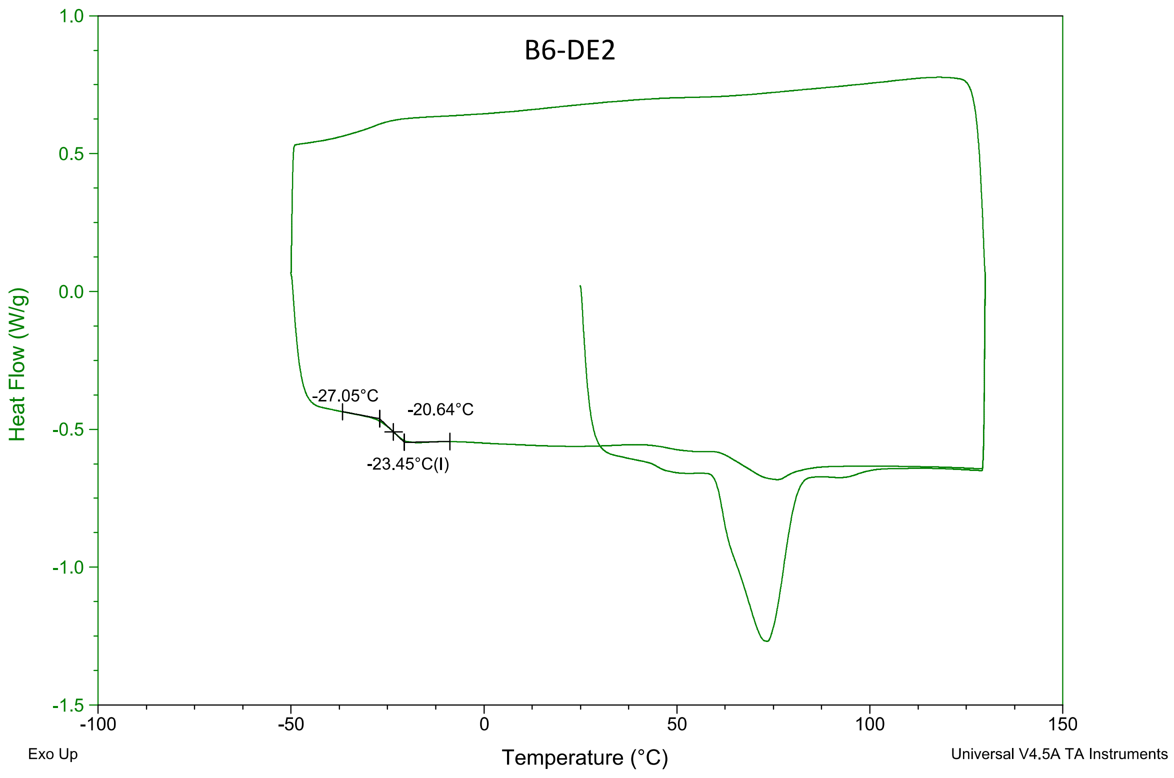

Figure S40. DSC thermograms of B6-DE2 for T_g evaluation.

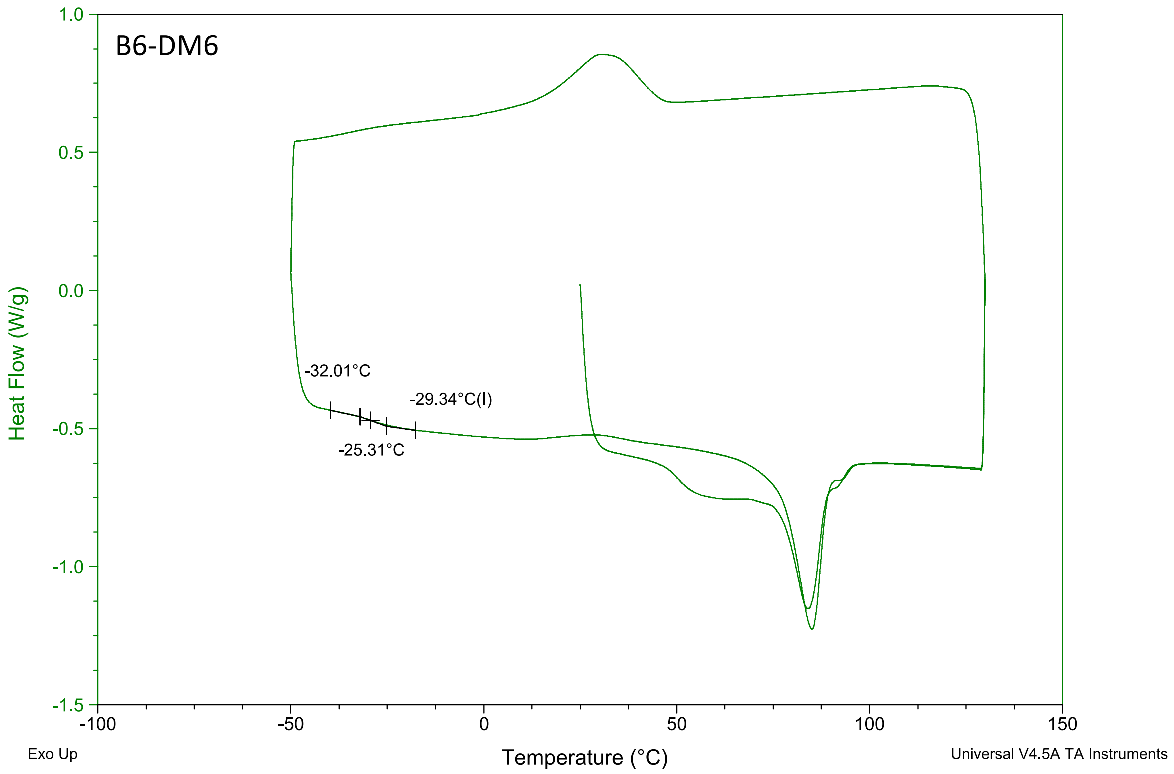

Figure S41. DSC thermograms of B6-DM6 for T_g evaluation.

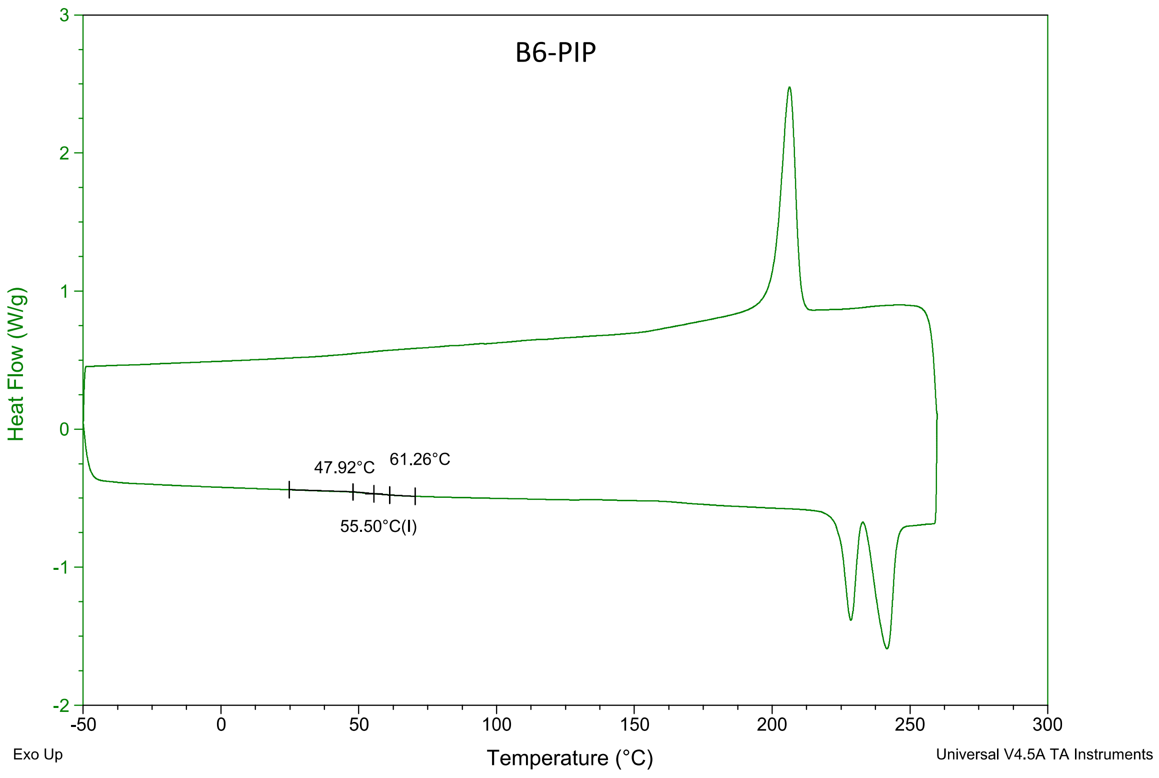

Figure S42. DSC thermograms of B6-PIP for T_g evaluation.

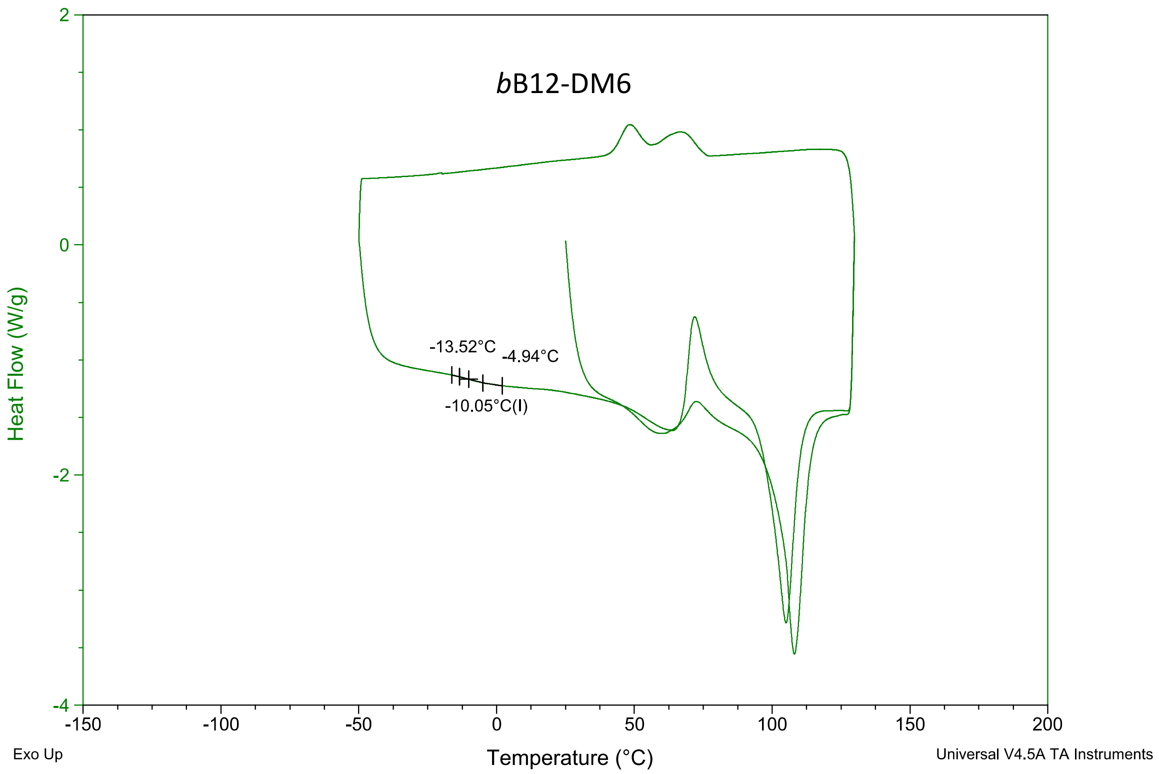

Figure S43. DSC thermograms of *b*B12-DM6 for T_g evaluation.

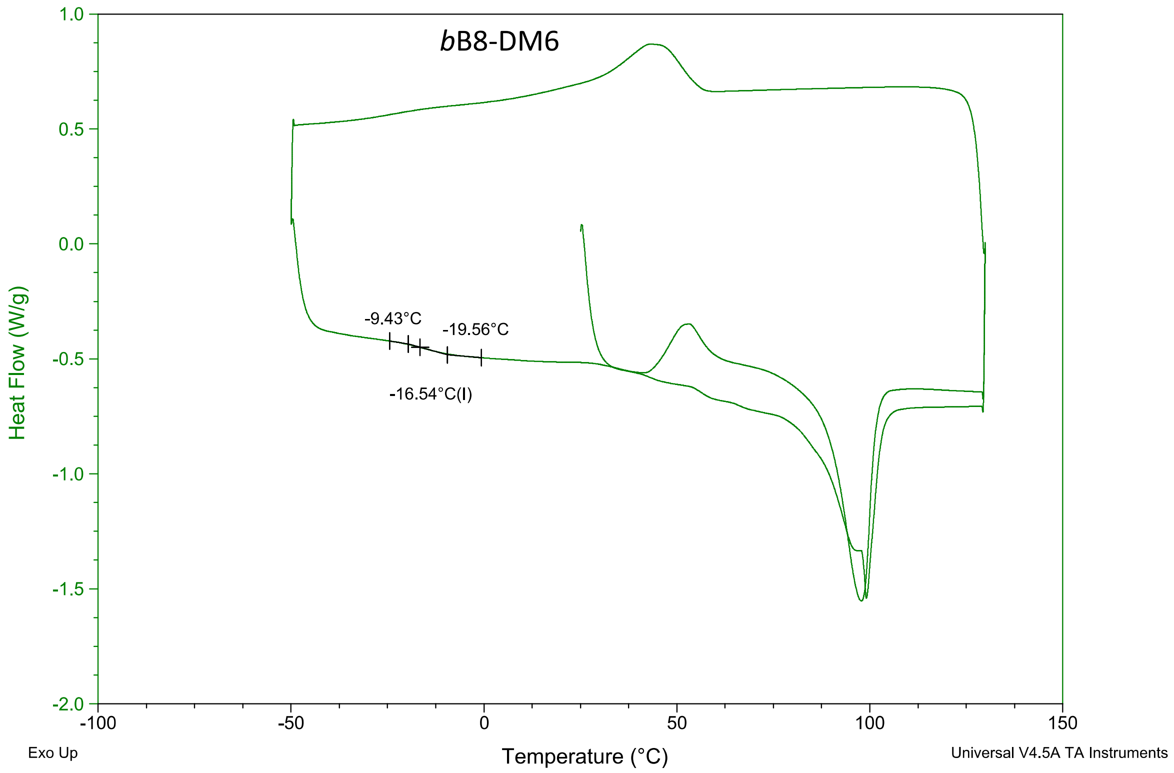

Figure S44. DSC thermograms of *b*B8-DM6 for T_g evaluation.
